# SPTBN1 abrogates renal clear cell carcinoma progression via glycolysis reprogramming in a GPT2-dependent manner

**DOI:** 10.1186/s12967-022-03805-w

**Published:** 2022-12-16

**Authors:** Jiajin Wu, Chenkui Miao, Yuhao Wang, Songbo Wang, Zhongyuan Wang, Yiyang Liu, Xiaoyi Wang, Zengjun Wang

**Affiliations:** 1 Department of Urology, The First Affiliated Hospital of Nanjing Medical University/Jiangsu Province Hospital, No. 300 Guangzhou Road, Nanjing, 210029 China; 2 Core Facility Center, The First Affiliated Hospital of Nanjing Medical University/Jiangsu Province Hospital, No. 300 Guangzhou Road, Nanjing, 210029 China

**Keywords:** SPTBN1, Renal clear cell carcinoma, Prognosis, Glycolysis, GPT2, Biomarker, Metabolic reprogramming

## Abstract

**Background:**

Renal clear cell carcinoma (ccRCC) is the most prevalent tumors worldwide. Discovering effective biomarkers is essential to monitor the prognosis and provide alternative clinical options. SPTBN1 is implicated in various cancerous processes. However, its role in ccRCC remains unelucidated. This study intends to explore the biological function and mechanism of SPTBN1 in ccRCC.

**Methods:**

Single-cell and bulk RNA-seq, tissue microarray, real-time quantitative PCR, and western blotting were applied to verify the expression and predictive value of SPTBN1 in ccRCC. Gain or loss of functional ccRCC cell line models were constructed, and in vitro and in vivo assays were performed to elucidate its tumorigenic phenotypes. Actinomycin D experiment, RNA immunoprecipitation (RIP), specific inhibitors, and rescue experiments were carried out to define the molecular mechanisms.

**Results:**

SPTBN1 was down-regulated in ccRCC and knockdown of SPTBN1 displayed a remarkably oncogenic role both in vitro and in vivo; while overexpressing SPTBN1 reversed this effect. SPTBN1 mediated ccRCC progression via the pathway of glutamate pyruvate transaminase 2 (GPT2)-dependent glycolysis. The expression of GPT2 was significantly negatively correlated with that of SPTBN1. As an RNA binding protein SPTBN1, regulated the mRNA stability of GPT2.

**Conclusion:**

Our research demonstrated that SPTBN1 is significantly down-regulated in ccRCC. SPTBN1 knockdown promotes ccRCC progression via activating GPT2-dependent glycolysis. SPTBN1 may serve as a therapeutic target for the treatment of ccRCC.

**Supplementary Information:**

The online version contains supplementary material available at 10.1186/s12967-022-03805-w.

## Introduction

Renal cell carcinoma (RCC), a common urinary malignancy worldwide, is estimated to bring 77,410 new cases in China and 79,000 in USA in 2022 [1–3]. Among the three subtypes of RCC [4], ccRCC makes up about 70% [5]. Although treated with various new diagnostic and surgical strategies, around 1/3 of patients still undergo local recurrence or distant metastasis [6, 7]. Targeted drugs, including tyrosine kinase inhibitors (TKIs), and mTOR have exhibited evident effectiveness as first-line treatment for metastatic RCC [8–10]. However, the diagnosis is still difficult, and the survival is poor [11–13]. New biomarkers and their molecular mechanisms should be discovered to design better treatment options for ccRCC.

The genes in the Spectrin-family were first discovered in erythrocytes in 1968 by Marchesi and Steersand, including erythroid Spectrin and nonerythroid Spectrin [14]. βII Spectrin (SPTBN1) functions as the most common dynamic intracellular protein of non-erythrocyte Spectrin, a cytoskeletal protein from the F-actin cross-linking protein superfamily presenting in all nucleated cells [15, 16]. As a Spectrin-family gene, SPTBN1 is an essential component of conventional βII Spectrin isoforms. SPTBN1 also serves to maintain cellular structure, function, and cycle [17, 18]. Aberrant SPTBN1 expression drives the progression and metastasis of multiple human malignancies. In breast cancer, SPTBN1 down-regulates miR-21 to suppress the epithelial-mesenchymal transition (EMT), thus inhibiting the proliferation and metastasis [19]. The deletion of SPTBN1 activates abnormal Wnt/β-catenin signaling pathway, thereby manifesting the characteristics of liver cancer stem cells [20]. As a novel tumor suppressor, SPTBN1 expression is dysregulated in hepatocellular carcinoma [20–22], pancreatic cancer [23, 24], colorectal cancer [25], ovarian cancer [26] and lung cancer [27]. SPTBN1 suppresses tumor progression via Wnt/β-catenin, JAK/STAT3 and TGF-β/SMAD pathways [28, 29]. However, the biological function and underlying mechanism of SPTBN1 in ccRCC have never been elucidated.

Metabolic reprogramming is a hallmark of cancer. ccRCC can be characterized as a “metabolic disease”, due to the excessive metabolic activity during its development [30, 31]. To our knowledge, von Hippel-Lindau (VHL) gene harbors the most frequent mutations in ccRCC, while its absence or alteration can stabilize the activities of hypoxia-inducible factor (HIF) family members [32], thereby activating a large repertoire of genes involved in glucose uptake and glycolytic metabolism pathways [33]. To obtain the energy required for rapid proliferation, tumor cells consumed glucose and lactate through boosting glycolysis, even under aerobic conditions [34, 35]. Glycolysis process involves various enzymes, including SLC2A1, PKM, HKI, HKII, GPT2, and LDHA, all recognized as potential therapeutic targets [36, 37]. Targeting glycolysis with 2-deoxy-D-glucose can inhibit ccRCC proliferation and sensitize ccRCC cells to TKIs [38]. Likewise, overexpression of PFKFB3 enhances glycolysis to facilitate ccRCC proliferation [39]. These research fruits have highlighted the possibility of targeting glycolysis to treat ccRCC.

In the present study, we aimed to investigate the aberrant expression pattern of SPTBN1 in ccRCC and its tumor-suppressive role, as well as its interaction with downstream GPT2 to suppress tumor-required glycolysis. Our data may value SPTBN1 as a biomarker for the early diagnosis and precise treatment of ccRCC.

## Materials and methods

### Clinical samples collection

ccRCC and adjacent normal renal samples were obtained by radical nephrectomy from the First Affiliated Hospital of Nanjing Medical University (Jiangsu Province Hospital) between 2005 and 2018. Detailed clinicopathological information of involved patients was listed in Table 3. All diagnoses were confirmed by senior pathologists independently. Informed consent was provided by all patients. The study design and protocol were approved by the ethics committee of the First Affiliated Hospital of Nanjing Medical University (Jiangsu Province Hospital).

### Tissue microarray (TMA) and immunohistochemistry (IHC)

IHC was performed as previously described [40, 41]. Briefly, the primary antibodies were diluted as follows: anti-SPTBN1 (1:150, Abcam, USA) and anti-GPT2 (1:200, Proteintech, China). TMA was constructed to analyze a total of 180 ccRCC samples. To assess the degree of protein staining expression level, TMA staining signals were quantified using H-score system [42] (H-Score = ∑(I × Pi), I = intensity of staining, and Pi = percentage of stained tumor cells, ranging from 0 to 300). The expression level of protein was separately and independently assessed by two experienced pathologists blinded to the clinical outcomes. According to the average expression level of IHC, the SPTBN1 in ccRCC patients was considered as low expression (H-score < 70) and high expression (H-score ≥ 70). The GPT2 was considered as low expression (H-score < 60) and high expression (H-score ≥ 60).

### Cell culture and transfection

RCC cell lines (786-O, 769-P, ACHN, Caki-1) and human renal tubular epithelial cell line (HK-2) were purchased from the Type Culture Collection of the Chinese Academy of Sciences (Shanghai, China) and cultured in RPMI 1640 (786-O, 769-P), McCoy's 5A (Caki-1), DMEM (ACHN) and DMEM/F12 (HK-2) (Gibco, Thermo Fisher Scientific, USA) containing 10% fetal bovine serum (FBS) and 1% penicillin/streptomycin (Gibco, Thermo Fisher Scientific, USA). The lentiviral vectors containing short hairpin RNAs targeting SPTBN1 (shSPTBN1) and negative control (shNC) were constructed and transfected.2-Deoxy-D-glucose (2-DG; Selleck, China), a glycolysis inhibitor, was applied to inhibit aerobic glycolysis. Cells were transfected with small interfering RNA (siRNA) and overexpression SPTBN1 plasmid (oeSPTBN1) using Lipofectamine 3000 (Invitrogen, Thermo Fisher Scientific, USA).

### RNA isolation and real-time quantitative PCR (qRT-PCR)

Total RNA was extracted using TRIzol Reagent (Sigma-Aldrich, Merck, Germany). HiScript III All-in-one RT SuperMix (Vazyme, China) was used for cDNA synthesis according to the manufacturer’s instructions. QRT-PCR was performed with SYBR qPCR Master Mix (Vazyme, China) using StepOne Plus (Applied Biosystems, USA) and LightCycler 480 PCR instrument (Roche Diagnostics, Switzerland). The primers and siRNA Oligo used were listed in Additional file 1: Table S1.

### Western blotting (WB) assay

Total proteins were extracted using RIPA buffer supplemented with Halt protease and phosphatase inhibitor cocktail (Thermo Fisher Scientific, USA). Then, total 20 μg of protein was resolved using SDS-PAGE gels (4%-12%) and transferred onto polyvinylidene fluoride (PVDF) membranes (Millipore, USA). The membranes were blocked in TBST containing 5% skim milk powder for 2 h at room temperature. The membranes were incubated with the primary antibodies overnight at 4 °C, then with the secondary antibodies conjugated to horseradish peroxidase (HRP) for 1.5 h at room temperature, respectively. Band signals were detected by chemiluminescence system (Bio-Rad, USA).

### Cell proliferation and colony formation assay

Pretreated cells were counted and seeded into 96-well plates. Cell proliferation was measured using the CCK-8 Cell Counting Kit (Vazyme, China). The absorbance was measured at 450 nm with a microplate reader, following a 2-h incubation at 37 °C.

For the colony formation assay, pretreated cells were seeded into 6-well plates and incubated for 10–15 days. Colonies were fixed in 4% paraformaldehyde for 20 min and stained with 0.1% crystal violet for further analysis.

### Cell cycle determination

Cells were labeled using PI/RNase Staining Buffer (BD Biosciences, USA) according to the manufacturer’s protocol and followed by flow cytometry (BD Biosciences, USA). The results were further analyzed using Modfit software.

### Transwell migration and invasion assay

In the migration assay, pretreated cells were seeded into the upper 24-well Transwell chambers with serum-free medium. Matrigel medium (Corning, USA) was applied for invasion assay. The medium containing 20% FBS was added to the bottom chamber. After incubation at 37 °C for 24 or 36 h, the cells were fixed in 4% paraformaldehyde, stained with 0.1% crystal violet, and captured in five randomly selected fields by a microscope. All the experiments were repeated for at least three times.

### Tumor xenograft in vivo experiments

All the mouse experiments were approved by the Institutional Animal Care and Use Committee (IACUC) of Nanjing Medical University (No. IACUC-1908003). Briefly, total 2 × 10^7^ 786-O cells with stably knockdown-SPTBN1 (shSPTBN1) and negative control cells were collected and suspended with PBS and Matrigel (1:1, Corning, USA), then subcutaneously injected into 4-week-old female BALB/c nude mice. The tumor formation and volume were measured every other day. The formula of tumor volume was calculated as follows: Tumor volume = length * width^2^ / 2. Xenograft tumor samples were fixed in 4% paraffin for further research.

### RNA stability determination

786-O and Caki-1 cells were treated with 5 μg/mL Actinomycin D (Selleck, China) for 0, 2, 4, 6, 8, or 10 h. Total RNA was harvested for qRT-PCR experiments. The level of GPT2 transcript was normalized to that of β-actin control, and the relative half-life of GPT2 was calculated using GraphPad software.

### RNA immunoprecipitation (RIP)

RNA immunoprecipitation (RIP) assay was performed with Magna RIP RNA-Binding Protein Immunoprecipitation Kit (Millipore, USA) according to manufacturer’s protocol. Briefly, a total of 2*10^7^ 786-O and Caki-1 cells were lysed in RIP lysis buffer, then incubated with magnetic beads conjugated with anti-SPTBN1 or anti-Rabbit IgG antibodies for 6 h at 4 °C temperature. After centrifugation and washing, the supernatant was treated with Proteinase K. Finally, the immunoprecipitated RNA was purified and extracted using TRIzol Reagent for further qRT-PCR analysis.

### Library preparation and transcriptome sequencing

786-O shNC and 786-O shSPTBN1 cells were applied for RNA-sequencing in biological duplicates. For each sample, 1.5 μg of RNA was generated for library preparation using NEBNext Ultra RNA Library Prep Kit for Illumina (NEB, USA). The library preparation was accomplished on an Illumina Hiseq-4000 platform (Allwegene, Beijing, China). Raw data in fastq format were processed for quality control and further analyzed using “DESeq” R package [43, 44] (Benjamini and Hochberg’s algorithm). Genes with adjusted *P*-value (*q*-value) < 0.05 were considered as differentially expressed.

### Dual-luciferase reporter assay

786-O and Caki-1 ccRCC cells were transfected with pEZX-FR02, a luciferase reporter containing wild-type or mutated GPT2-3’UTR region. The ratios of firefly and Renilla luciferase activities were calculated after 48 h of transfection using Dual-Luciferase Assay System (Promega, USA).

### Glucose consumption, lactate secretion, and ATP concentration measurement

Assay kits (Nanjing Jiancheng Bioengineering, China) were used to measure the glucose concentrations, lactic acid production, and ATP level in 786-O and Caki-1 ccRCC cell lines. Concentration was calculated and normalized to cell number.

### Bioinformatics analysis of in silico database

TCGA-KIRC cohort, GSE6344, GSE40435, GSE46699, GSE53757, GSE66270, GSE105261, together with single-cell RNA-seq GSE152938 and GSE156632 datasets were employed in this study [45–54]. Cancer Cell Line Encyclopedia (CCLE), CPTAC and UALCAN databases were also applied [55–57]. The proteogenomic expression data in Chinese ccRCC patients were downloaded from the supplementary materials of Qu [58]. For single-cell RNA-seq analysis, R package “Seurat” was used to process data [54, 59–61]. The cells expressing > 20% mitochondria-related genes, > 5% of hemoglobin reads, less than 500 genes, or greater than 10,000 genes were filtered out, then we normalized and rescaled the expression matrix. Next, cell clusters were obtained through the UMAP method. Finally, we used the “SingleR” package to annotate the cell clusters [62].

### Statistical analysis

Statistical analysis was run on SPSS and R software. Data were expressed with the means ± SD. The independent t-test was used to compare continuous variables that exhibited normal distributions. The Wilcoxon test was used to compare the continuous variables that were not normally distributed. Kaplan–Meier survival analysis and Cox hazard regression model were employed to sift out factors predicting the overall survival, disease-specific survival, disease-free interval, and progression-free interval prognostic factors. Correlations analysis was performed by Pearson and Spearman methods. All experiments were repeated at least three times. All statistical tests were two-sided, and *P* < 0.05 was considered statistically significant. ns: No significant; *:*P* < 0.05; **:*P* < 0.01; ***:*P* < 0.001; ****:*P* < 0.0001.

## Results

### Expression profile of Spectrin-family genes in ccRCC

**Fig. 1:**
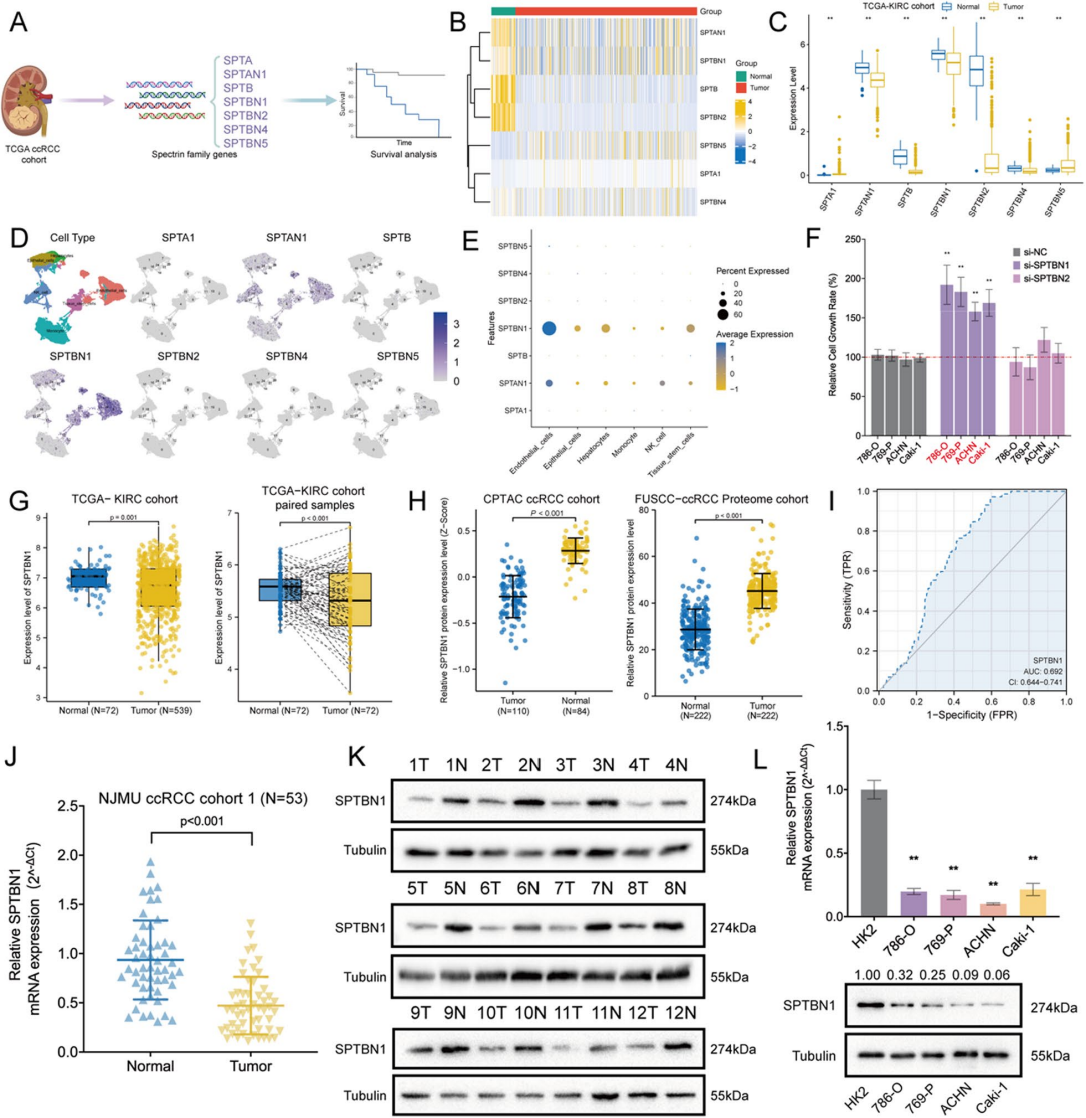
SPTBN1 was down-regulated in ccRCC. **A** The flowchart of our screening of Spectrin-family genes. **B** The heatmap of Spectrin-family genes expression patterns. **C** Boxplot showed the expression levels of Spectrin-family genes in TCGA-KIRC database. **D** The distribution of Spectrin-family genes from single-cell RNA-seq. **E** Dot plot of the expression of Spectrin-family genes in each cell. **F** Relative growth rate of RCC cell lines after transfection with small interfering RNA of SPTBN1 and SPTBN2. **G** The mRNA expression level of SPTBN1 in paired and unpaired samples from the TCGA-KIRC database. **H** The protein expression level of SPTBN1 in the CPTAC database and FUSCC-ccRCC Proteome cohort. **I** The receiver operating characteristic (ROC) curve of SPTBN1 among the TCGA-KIRC cohort. **J** qRT-PCR confirmed SPTBN1 was down-regulated in ccRCC tumor samples. **K** Western blot illustrated the expression of SPTBN1 in ccRCC clinical samples. **L** The expression of SPTBN1 in RCC cell lines by qRT-PCR and WB. (**: *P* < 0.05)

Seven Spectrin-family genes with significantly abnormal expression profiles in ccRCC tissues were screened out from the TCGA-KIRC, along with the clinicopathological parameters of related patients [15, 16, 28, 29]. The flowchart of screening process was illustrated in Fig. 1A. Our results confirmed that SPTA1 and SPTBN5 were up-regulated in ccRCC tissues, while SPTAN1, SPTB, SPTBN1, SPTBN2, and SPTBN4 were down-regulated in ccRCC tissues (Fig. 1B, C). Meanwhile, we analyzed the distribution of Spectrin-family genes using datasets of single-cell RNA-sequencing (Additional file 1: Figure S1A-E). SPTBN1 was mainly enriched in endothelial cells and tissue stem cells, and SPTBN2 in endothelial cells and natural killer cells (Fig. 1D, E). Then, we conducted univariate and multivariate cox regression analyses to assess the prognostic value of Spectrin-family genes. Among these candidate genes, only SPTBN1 and SPTBN2 were significantly associated with overall survival (OS), disease-specific survival (DSS), and progression survival interval (PFI) (Additional file 1: Figure S2A–C). In addition, the correlations between Spectrin-family genes were analyzed (Additional file 1: Figure S3A). Functional enrichment analysis demonstrated that Spectrin-family genes were mainly involved in the interaction between L1 and Ankyrins (Additional file 1: Figure S3B). After targeting small interfering RNA to SPTBN1 and SPTBN2, we identified SPTBN1 as a candidate target in ccRCC among Spectrin-family genes (Fig. 1F). Finally, our pan-cancer analysis discovered that SPTBN1 was significantly down-regulated in the tissue samples of most cancer subtypes, compared to that in adjacent normal samples (Additional file 1: Figure S4A). Based on the Cancer Cell Line Encyclopedia (CCLE) dataset, we observed that SPTBN1 was highly expressed in kidney cancer, compared to that in most of other solid tumors [63] (Additional file 1: Figure S4B). These findings indicated that SPTBN1 might act as a tumor suppressor role in ccRCC.

### SPTBN1 expression was down-regulated in ccRCC

To further investigate the tumor-suppressing role of SPTBN1 in ccRCC, firstly we detected the expression of SPTBN1 in multiple databases. The results showed that SPTBN1 was significantly decreased in both paired and unpaired samples from the TCGA-KIRC cohort (*P* < 0.001; Fig. 1G). This expression patterns was further validated in external GEO databases, including GSE66270, GSE105261, GSE6344, GSE40435, GSE46699 and GSE53757. (Additional file 1: Figure S5A–F). CPTAC and FUSCC proteome databases also demonstrated that the protein level of SPTBN1 was downregulated in ccRCC tissues (*P* < 0.001; Fig. 1H).

We further explored the expression of SPTBN1 in clinical ccRCC samples by IHC, qRT-PCR and WB. IHC staining results confirmed that the expression of SPTBN1 was remarkably decreased in tumor tissues (Additional file 1: Figure S5G). Besides, the receiver operating characteristic (ROC) curve analysis showed a high diagnostic value of SPTBN1 for ccRCC (AUC:0.692; 95% CI = 0.644–0.741; Fig. 1I). Moreover, qRT-PCR and WB also demonstrated the same results (NJMU ccRCC cohort 1: N = 53; Fig. 1J, K). In accordance with the findings in TCGA and GEO datasets, SPTBN1 expression was significantly down-regulated in ccRCC cell lines (786-O, 769-P, ACHN, and Caki-1) at both mRNA and protein levels, compared with that in renal tubular normal epithelial cell line HK-2 (*P* < 0.05; Fig. 1L).

### SPTBN1 down-regulation predicted an unfavorable survival of ccRCC patients

**Fig. 2:**
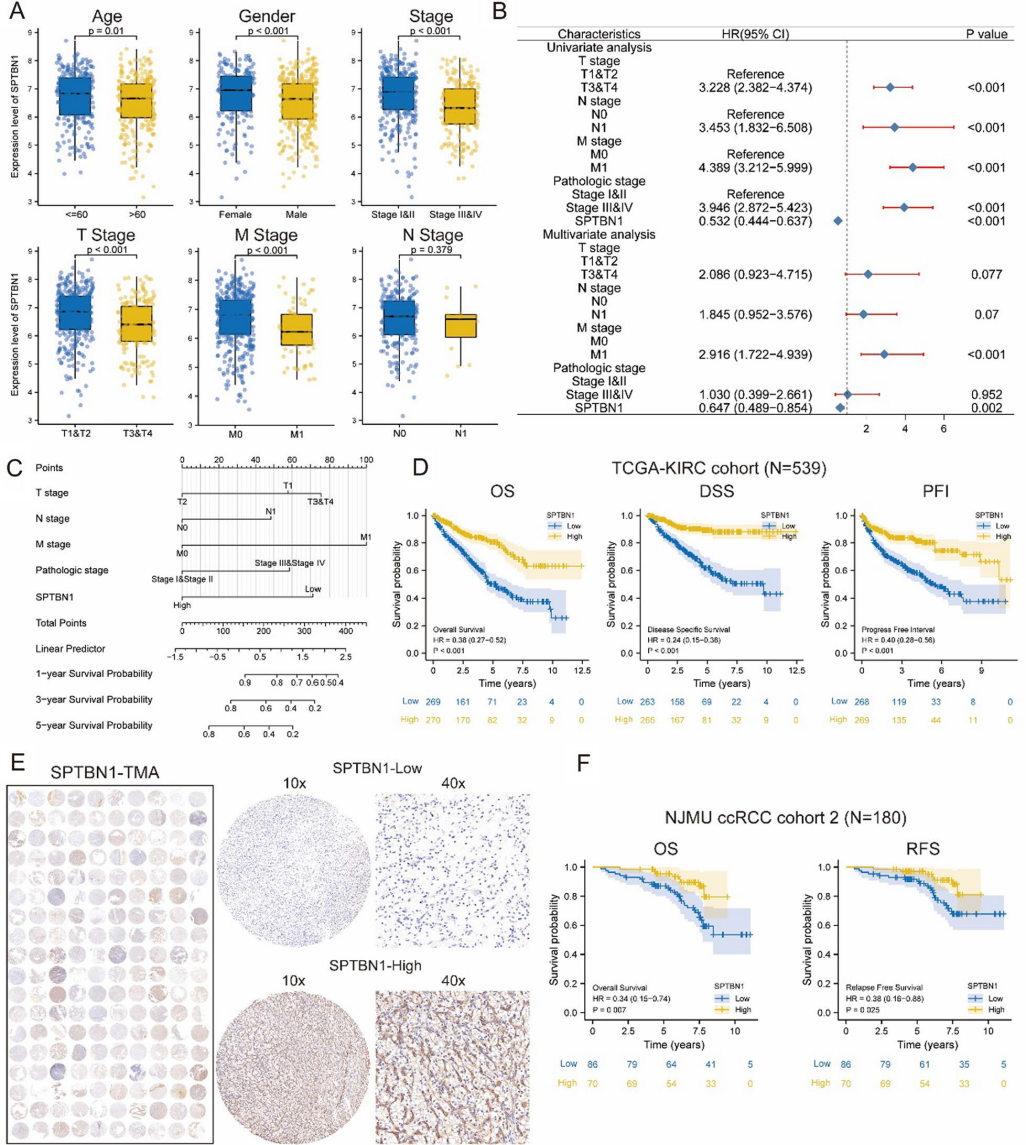
SPTBN1 down-regulation predicted advanced stage and poor survival of ccRCC. **A** Down-regulated SPTBN1 mRNA expression level was correlated with an advanced ccRCC stage **B** Univariate and multivariate cox regression analysis revealed SPTBN1 as an independent prognostic factor for ccRCC patients’ survival. **C** The nomogram for SPTBN1 and other clinical information. **D** Low SPTBN1 expression level predicted worse OS, DSS and PFI in the TCGA-KIRC cohort. **E** Landscape of tissue microarray staining for SPTBN1 in the NJMU ccRCC cohort 2 (N = 180). **F** The Kaplan–Meier survival curves of patients stratified by SPTBN1 expression level

**Fig. 3:**
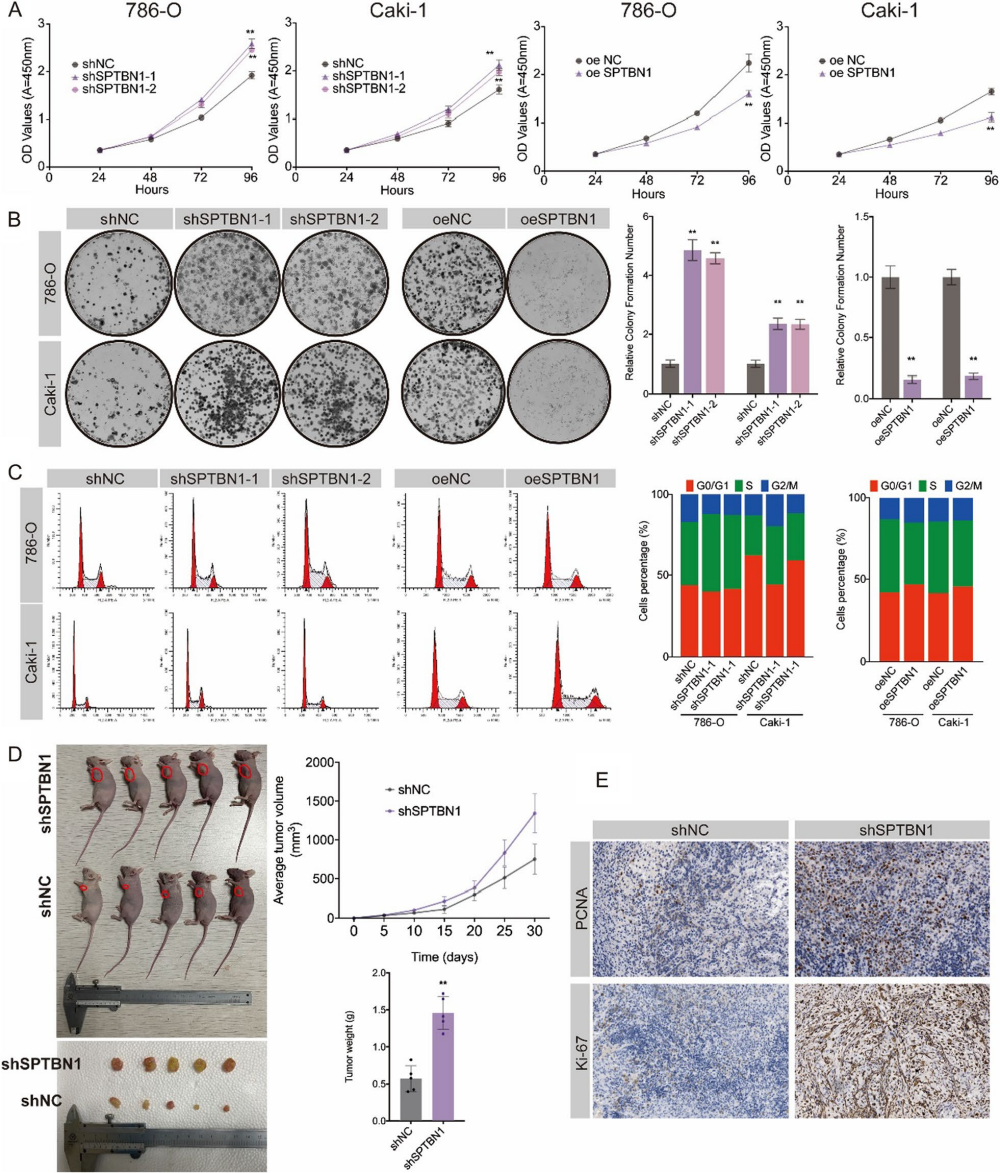
Knockdown SPTBN1 promoted inhibited the proliferation of ccRCC cells in vitro and in vivo. **A** Cell growth after knockdown or overexpression SPTBN1 at indicated time points. **B** Colony formation assay of RCC cells after knockdown or overexpression SPTBN1. **C** Cell cycle distribution in control, knockdown SPTBN1 and overexpression SPTBN1 RCC cells. **D** Knockdown SPTBN1 promoted ccRCC cell proliferation in vivo. Tumor volume was monitored every other day.** E** IHC of PCNA, and Ki-67 derived from shSPTBN1 786-O cells. (**: *P* < 0.05)

We observed that low expression of SPTBN1 was positively correlated with high age, TNM stage, and tumor grade (*P* < 0.05, Fig. 3A; Table 1). Meanwhile, the analysis based on CPTAC and FUSCC proteome databases revealed low SPTBN1 expression in high-grade ccRCC patients (*P* < 0.05; Additional file 1: Figure S6A, B). In univariate and multivariate cox regression analyses, SPTBN1 served as an independent predictive factor for the survival of ccRCC patients (Univariate: HR = 0.532, 95% CI = 0.444 − 0.637, *P* < 0.001; Multivariate: HR = 0.647, 95% CI = 0.489–0.854, *P* = 0.002; Fig. 2B). We also examined the correlation between SPTBN1 and survival in subgroups of different clinicopathological characteristics, discovering that a low expression of SPTBN1 might predict a poor prognosis (Additional file 1: Figure S7A–D). The logistic regression analysis was adopted to detail the correlativity between SPTBN1 expression and clinicopathological characteristics (Table 2). Moreover, a nomogram based on TNM stage and SPTBN1 was developed to predict the 1-, 3-, and 5-year OS of each ccRCC patient (Fig. 2C). Kaplan–Meier analysis further indicated that a low SPTBN1 expression was correlated with worse overall survival, disease-specific survival, and progression-free survival in the TCGA-KIRC cohort (Fig. 2D). According to SPTBN1 expression in our tissue microarray from NJMU TMA cohort (N = 180), we divided the patients into high-SPTBN1 and low-SPTBN1 expression subgroups (Fig. 3E; Table 3). The results showed that a low SPTBN1 expression was associated with poor overall survival and relapse-free survival (*P* < 0.01; Fig. 3F). Simultaneously, we observed that SPTBN1 was predominantly down-regulated in ccRCC patients with advanced clinicopathological characteristics, which was consistent with the results of TCGA-based analysis (Table 3). Taken together, SPTBN1 possessed a high prognostic value for ccRCC patients.

**Table 1:** Correlation between SPTBN1 expression and clinicopathological characteristics in the TCGA ccRCC cohort

Characteristics	Low expression of SPTBN1	High expression of SPTBN1	*P*-Value
Total	269	270	
Age (%)			0.043
< = 60	122 (22.6%)	147 (27.3%)	
> 60	147 (27.3%)	123 (22.8%)	
Gender (%)			0.002
Female	75 (13.9%)	111 (20.6%)	
Male	194 (36%)	159 (29.5%)	
Stage (%)			< 0.001
Stage I	102 (19%)	170 (31.7%)	
Stage II	32 (6%)	27 (5%)	
Stage III	75 (14%)	48 (9%)	
Stage IV	58 (10.8%)	24 (4.5%)	
T stage (%)			< 0.001
T1	106 (19.7%)	172 (31.9%)	
T2	42 (7.8%)	29 (5.4%)	
T3	113 (21%)	66 (12.2%)	
T4	8 (1.5%)	3 (0.6%)	
N stage (%)			0.124
N0	125 (48.6%)	116 (45.1%)	
N1	12 (4.7%)	4 (1.6%)	
M stage (%)			< 0.001
M0	199 (39.3%)	229 (45.3%)	
M1	55 (10.9%)	23 (4.5%)

**Table 2:** Logistic regression analysis for SPTBN1 expression and clinicopathological characteristics

Characteristics	Total(N)	Odds Ratio (OR)	*P*-Value
Age (> 60 vs. < = 60)	539	0.694 (0.494–0.974)	0.035
Gender (male vs. female)	539	0.554 (0.385–0.793)	0.001
T stage (T2&T3&T4 vs. T1)	539	0.371 (0.261–0.524)	< 0.001
N stage (N1 vs. N0)	257	0.359 (0.098–1.064)	0.083
M stage (M1 vs. M0)	506	0.363 (0.212–0.605)	< 0.001
Stage (stage III&IV vs. stage I&II)	536	0.368 (0.256–0.527)	< 0.001

**Table 3:** Correlation between SPTBN1 expression and clinicopathological characteristics in the NJMU ccRCC cohort (N = 180)

Characteristics	Number of cases	SPTBN1 expression		*P*-Value
		Low	High	
Total	180	106	74	
Age(years)				
< 65	103	61	42	0.916
≥ 65	77	45	32	
Gender				
Male	99	58	41	0.927
Female	81	48	33	
T stage				
T1–T2	110	52	58	< 0.001
T3–T4	70	54	16	
Histological grade				
I–II	78	32	46	< 0.001
III–IV	102	74	28	
Tumor size(cm)				
< 4	115	51	64	< 0.001
≥ 4	65	55	10	
Survival State				
Alive	119	60	59	0.034
Dead	37	26	11	
Recurrence				
Yes	28	21	7	0.020
No	128	65	63	
GPT2 protein expression level				
High	99	67	32	0.008
Low	81	39	42	

### SPTBN1 inhibited the proliferation of ccRCC cells and malignant potential in vitro and in vivo

To further investigate the biological function of SPTBN1 in ccRCC, we successfully established SPTBN1-knockdown and SPTBN1-overexpression cell models (786-O and Caki-1) and validated by qRT-PCR and WB (Additional file 1: Figure S8A, B, *P* < 0.05). Cell counting kit-8 (CCK-8) assay indicated that SPTBN1 knockdown significantly increased cell proliferation ability (Fig. 3A). Colony formation assay was also employed to determine the long-term impact of SPTBN1 on cells proliferation. Colony formation was more evident in SPTBN1 knockdown group than in control group, while SPTBN1 overexpression reversed this change (Fig. 3B). Furthermore, flow cytometry showed that SPTBN1 knockdown increased the proportion of 786-O and Caki-1 cells in the S phase and decreased that in the G0-G1 phase, while SPTBN1 overexpression resulted in opposite effect (Fig. 3C). Additionally, Transwell migration and invasion assay demonstrated that SPTBN1 knockdown turned CCRCC cells more aggressive and metastatic (Additional file 1: Figure S9A).

In addition, to determine the influence of SPTBN1 in vivo, we established subcutaneously xenograft tumor models using nude mice (Fig. 3D). Knocking down SPTBN1 dramatically accelerated tumor growth, as quantified by tumor size and tumor volume (Fig. 3D). Accordingly, IHC experiments further revealed that Ki-67 and PCNA, indicators of proliferation, were notably up-regulated in tumors derived from shSPTBN1 786-O cells, indicating more rapid tumor growth (Fig. 3E). Collectively, SPTBN1 could retard the G1/S progression and suppress the proliferation of ccRCC cells both in vitro and in vivo.

### SPTBN1 deficiency reprogrammed glycolysis in ccRCC cells

**Fig. 4:**
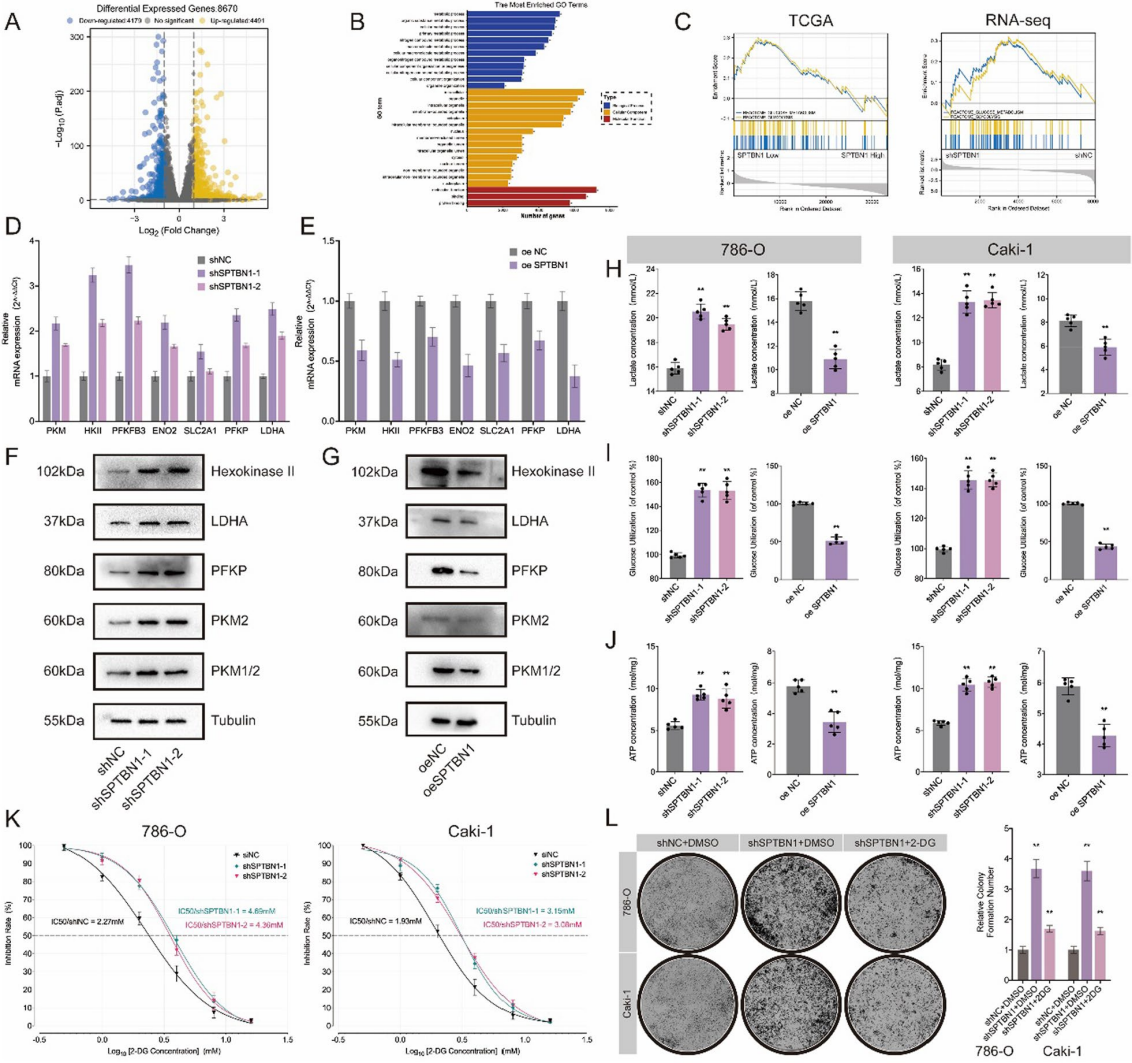
SPTBN1 knockdown activated ccRCC tumorigenesis through enhancing glycolysis. **A** RNA-seq differential expression analysis of SPTBN1-knockdown and control 786-O cells. Volcano illustrated differentially expressed genes. **B** Top enriched GO pathways in shSPTBN1 and shNC 786-O groups. **C** GSEA analysis showed Reactome Glucose Metabolism and Reactome Glycolysis pathways were significantly enriched. **D**, **E** qRT-PCR detected the expression of glycolysis-associated genes after SPTBN1 knockdown (**D**) or overexpression (**E**). **F**, **G** WB of glycolysis-associated genes after SPTBN1 knockdown (**F**) or SPTBN1 overexpression (**G**). **H** Lactate concentrations in RCC cells after SPTBN1 knockdown or overexpression. **I** Relative glucose consumption rates in RCC cells after SPTBN1 knockdown or overexpression. **J** ATP concentrations in RCC cells after SPTBN1 knockdown or overexpression. **K** SPTBN1 knockdown decreased the suppression rate of 2-DG in 786-O and Caki-1 cells. **L** 2-DG could partially rescue the malignant proliferation of SPTBN1-knockdown RCC cells in clonogenicity assays. (**: *P* < 0.05)

In order to clarify the underlying mechanism of SPTBN1 involved in RCC cell proliferation, we performed RNA-sequencing on shNC and shSPTBN1 786-O cells. A total of 4491 up-regulated genes and 4179 down-regulated genes were identified (Fig. 4A). Gene ontology (GO) functional enrichment analysis revealed their enrichment in metabolic process-related pathways (Fig. 4B). Subsequently, we performed gene set enrichment analysis (GSEA) using both TCGA-KIRC and our RNA-seq dataset. Interestingly, Reactome Glucose Metabolism and Reactome Glycolysis pathways were significantly associated with the biological function of SPTBN1 (Fig. 4C). To verify this hypothesis, we detected the expression level of rate-limiting enzymes involved in glycolytic process. Unexpectedly, glycolysis-associated genes (PKM, HKII, PFKFB3, ENO2, SLC2A1, PFKP, and LDHA) were dramatically up-regulated after SPTBN1 knockdown, whereas SPTBN1 overexpression reversed this influence (Fig. 4D–G). Consequently, we observed that knockdown of SPTBN1 significantly enhanced lactate concentrations (Fig. 4H), glucose consumption rates (Fig. 4I) and ATP levels (Fig. 4J) in ccRCC cell lines, while SPTBN1 overexpression substantially reversed this increase (Fig. 4H–J).

Ultimately, supplementation with 2 mM 2-deoxy-D-glucose (2-DG), a specific inhibitor of glycolysis, could partially block glycolysis-induced proliferation of ccRCC cells. SPTBN1 knockdown dramatically prevented 2-DG from suppressing 786-O and Caki-1 cells. The half-maximal inhibitory concentration (IC50) data revealed that SPTBN1 knockdown increased the IC50 value and weakened the sensitivity to 2-DG, but SPTBN1 overexpression displayed contradictory effects (Fig. 4K). Taken together, that 2-DG could partially repress the malignant proliferation of SPTBN1-knockdown ccRCC cells (Fig. 4L).

### SPTBN1 was negatively correlated with GPT2 expression

**Fig. 5:**
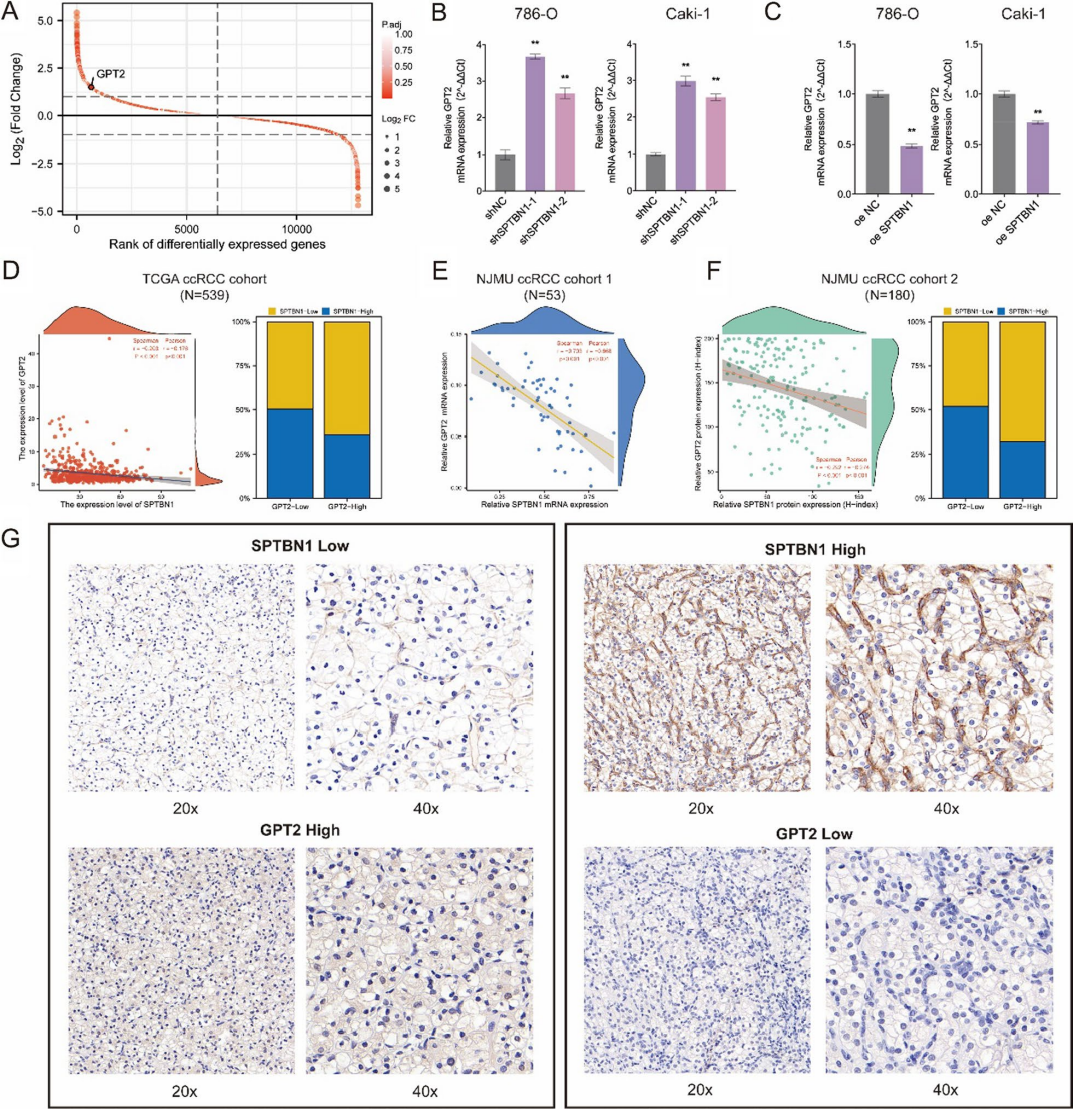
SPTBN1 expression was negatively correlated with GPT2 expression. **A** Distribution of genes with a significant difference in expression between SPTBN1-knockdown and control 786-O cells. **B** qRT-PCR results about the relative GPT2 mRNA expression levels in SPTBN1-knockdown and control RCC cells. **C** qRT-PCR results about the relative GPT2 mRNA expression levels in SPTBN1-overexpression and control RCC cells. **D** Correlation analysis for the mRNA expression levels of SPTBN1 and GPT2 in the TCGA-KIRC cohort. **E** Correlation analysis for the mRNA expression levels of SPTBN1 and GPT2 in the NJMU ccRCC cohort 1 (N = 53) by qRT-PCR. **F** Correlation analysis for the protein expression levels of SPTBN1 and GPT2 form the NJMU ccRCC cohort 2 (N = 180) by IHC of TMA. **G** Representative IHC images of TMA for SPTBN1 and GPT2 expression in ccRCC patients. (**: *P* < 0.05)

We next explored the downstream genes of SPTBN1 in the glycolytic pathway. We screened out the GPT2 gene based on log_2_FC (Fold Change) and *P*-value (Fig. 5A). qRT-PCR experiments further confirmed that GPT2 expression was significantly elevated in SPTBN1-knockdown 786-O and Caki-1 cells, while GPT2 mRNA levels fell significantly after SPTBN1 overexpression (Fig. 5B, C). The correlation analysis in the TCGA-KIRC database indicated that the expression levels of SPTBN1 and GPT2 were negatively correlated (Pearson: R = − 0.176, *P* < 0.001; Spearman: R = − 0.203, *P* < 0.001; Fig. 5D). In addition, the same results were observed in NJMU ccRCC cohort (N = 53; Pearson: R = − 0.703, P < 0.001; Spearman: R = − 0.668, *P* < 0.001; Fig. 5E). TMA immunohistochemical data also revealed a negative correlation between SPTBN1 and GPT2 (NJMU ccRCC TMA cohort2: N = 180; Pearson: R = − 0.274, *P* < 0.001; Spearman: R = − 0.292, *P* < 0.001; Fig. 5F, G).

### GPT2 up-regulation altered the oncogenicity in ccRCC cells

**Fig. 6:**
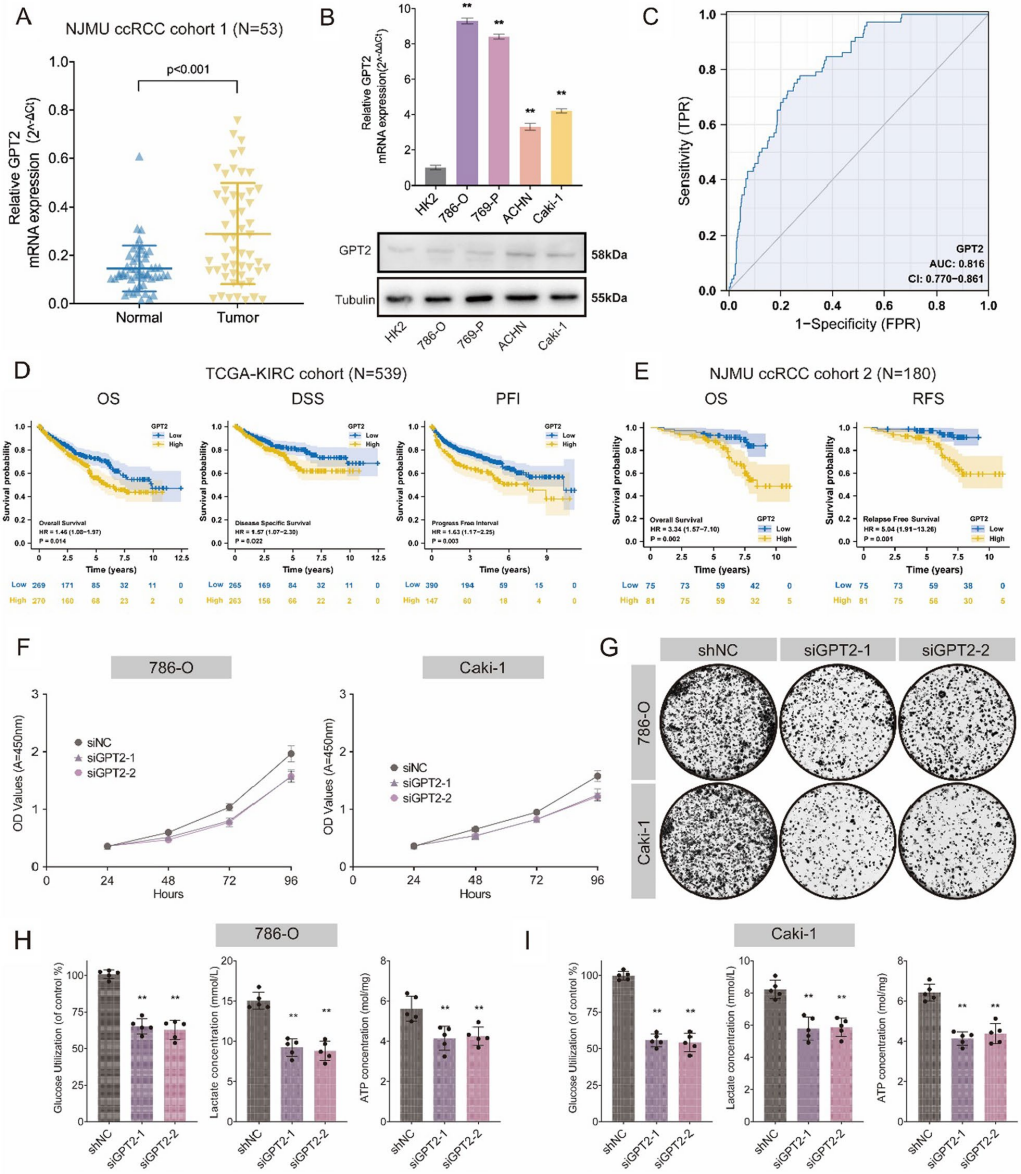
GPT2 up-regulation altered the oncogenicity in ccRCC cells. **A** qRT-PCR assay revealed GPT2 was up-regulated in ccRCC samples. **B** The mRNA and protein expression levels of GPT2 were up-regulated in ccRCC cell lines. **C** The receiver operating characteristic (ROC) curve of GPT2 in the TCGA-KIRC cohort. **D** High GPT2 expression level predicted worse OS, DSS and PFI in the TCGA-KIRC cohort. **E** Kaplan–Meier survival curves of patients stratified by GPT2 expression level in the NJMU ccRCC cohort 2 (N = 180). **F** Cell growth after GPT2 knockdown detected by CCK-8 assay. **B** Colony formation assay of RCC cells after GPT2 knockdown. **H** Lactate concentrations, glucose consumption rates and ATP concentration of 786-O cells after GPT2 knockdown. **I** Lactate concentrations, glucose consumption rates and ATP concentration of Caki-1 cells after GPT2 knockdown. (**: *P* < 0.05)

Subsequently, we evaluated the expression and prognostic value of GPT2 in ccRCC patients. qRT-PCR confirmed that GPT2 mRNA was obviously up-regulated in NJMU ccRCC cohort (N = 53, *P* < 0.001; Fig. 6A). Likewise, GPT2 up-regulation was observed in IHC (Additional file 1: Figure S10A). Meanwhile, qRT-PCR and WB showed that GPT2 expression increased significantly in various types of CCRCC cells, compared with that in HK2 cells (Fig. 6B). The ROC curve analysis showed an AUC of 0.816 (95%CI = 0.770–0.861; Fig. 6C). Kaplan–Meier survival analysis indicated that the prognosis (OS, DSS, and PFI) of patients with high GPT2 expression was significantly worse (OS: *P* = 0.014; DSS: *P* = 0.022; PFI: *P* = 0.003; Fig. 6D), demonstrating the excellent prognostic value of GPT2. According to our tissue microarray from NJMU TMA cohort (N = 180), we divided patients into high- and low-GPT2 expression subgroups. The results showed that high GPT2 expression predicted longer overall survival and relapse-free survival (*P* < 0.01; Fig. 6E). CCK-8 assay demonstrated that GPT2 knockdown significantly decreased cell proliferation (Fig. 6F). Colony-formation efficiency was reduced after GPT2 depletion (Fig. 6G). GPT2 knockdown significantly suppressed lactate concentration, glucose consumption rate and ATP level in ccRCC cell lines (Fig. 6H, I). In conclusion, GPT2, as a downstream gene of SPTBN1, promoted ccRCC progression via glycolysis.

### SPTBN1 regulated the stability of GPT2 mRNA by binding to its 3’-UTR regions

**Fig. 7:**
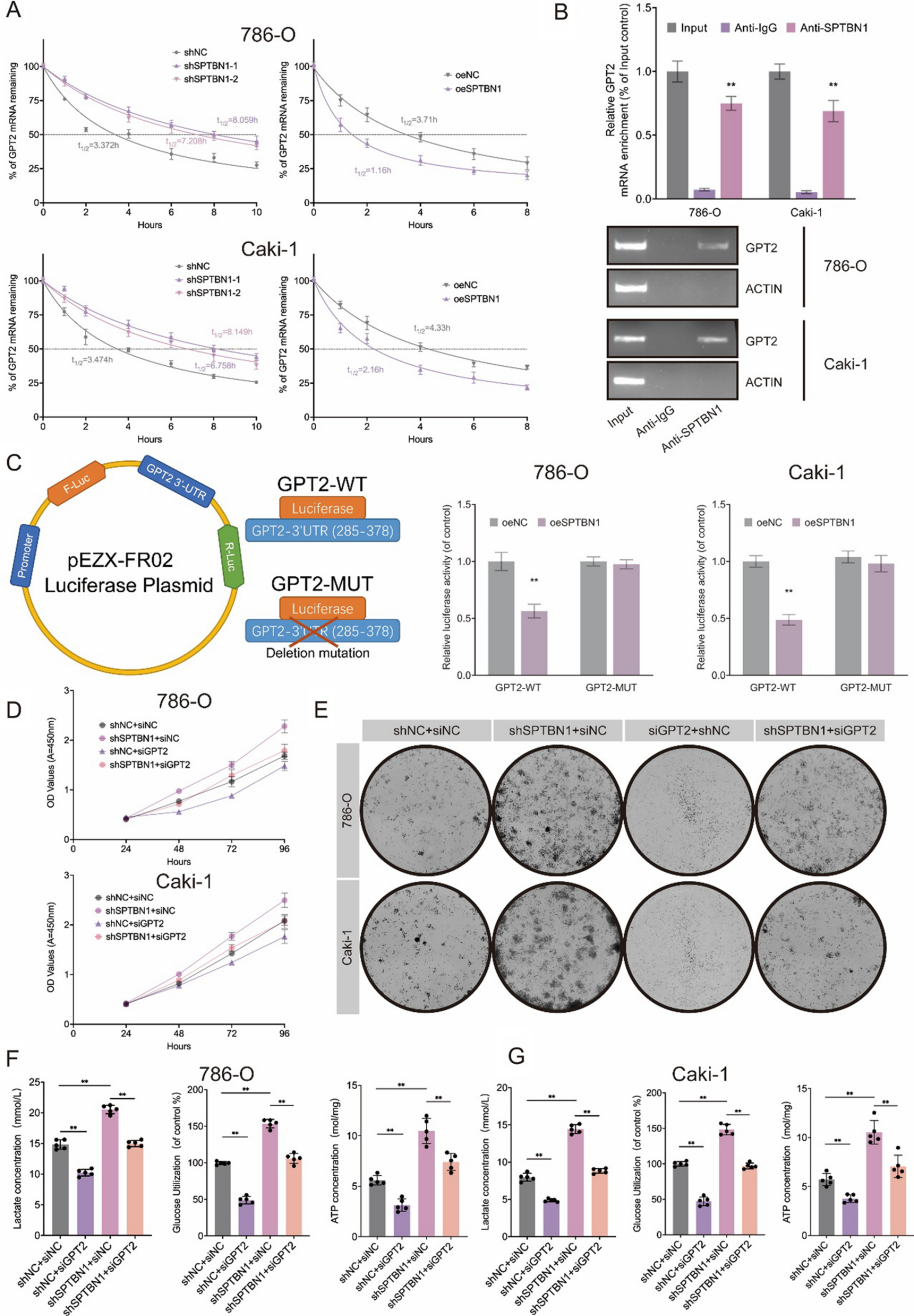
SPTBN1 regulated the stability of GPT2 mRNA by binding to its 3’-UTR regions. **A** The half-life of GPT2 mRNA expression was measured by qRT-PCR after SPTBN1knockdown or overexpression in ccRCC cells. **B** Relative GPT2 mRNA level in ccRCC cells by RIP-PCR. **C** Dual-luciferase reporter assay showed the influence of SPTBN1 and GPT2 with wild-type or mutation 3’-UTR binding sites. **D** Cell growth of SPTBN1-knockdown ccRCC cells transfected with GPT2 siRNA or negative control siRNA. **E** Clonogenicity assay of SPTBN1-knockdown ccRCC cells transfected with GPT2 siRNA or negative control siRNA. **F** Lactate concentrations, glucose consumption rates and ATP concentrations of SPTBN1-knockdown 786-O cells transfected with GPT2 siRNA or negative control siRNA. **G** Lactate concentrations, glucose consumption rates and ATP concentrations of SPTBN1-knockdown Caki-1 cells transfected with GPT2 siRNA or negative control siRNA. (**: *P* < 0.05)

Considering the interaction between SPTBN1 and GPT2 we ascertained above, we suspected whether depletion of SPTBN1 could alter GPT2 expression. The mRNA expression level of SPTBN1 was negatively correlated with that of GPT2, indicating SPTBN1 regulated GPT2 at the transcriptional level. Given that SPTBN1 is known as an RNA-binding protein, we hypothesized that SPTBN1 functions as an RBP binding to the 3’UTR region of GPT2 [64, 65]. Control and SPTBN1-deficient 786-O and Caki-1 cells were stimulated with actinomycin D for 0, 2, 4, 6, 8, and 10 h. Then, mRNA stability was monitored using qRT-PCR. This analysis revealed that SPTBN1-depleted cells predominantly contained more stable GPT2 mRNAs than control cells, whereas SPTBN1 overexpression suppressed GPT2 mRNA level in 786-O and Caki-1 cells (Fig. 7A). Congruently, RIP experiments in 786-O and Caki-1 (with SPTBN1 overexpression) cells further demonstrated that SPTBN1 could bind to GPT2 (Fig. 7B). In the dual-luciferase reporter assay, 786-O and Caki-1 CCRCC cells were transfected with pEZX-FR02 plasmid which contained mutated GPT2 3’-UTR region (GPT2-MUT) and wild type of GPT2 3’-UTR region (GPT2-WT). Compared with mutated GPT2 3’-UTR reporter plasmids, SPTBN1 could significantly reduce the luciferase activity of the wild-type GPT2 3’-UTR, which was in concordance with our hypothesis (Fig. 7C). These findings confirmed that SPTBN1 could function as an RNA-binding protein to regulate the stability of GPT2 mRNA by binding to its 3’-UTR regions.

### Silencing GPT2 can reversed the malignant proliferation of ccRCC cells with SPTBN1 deletion

**Fig. 8:**
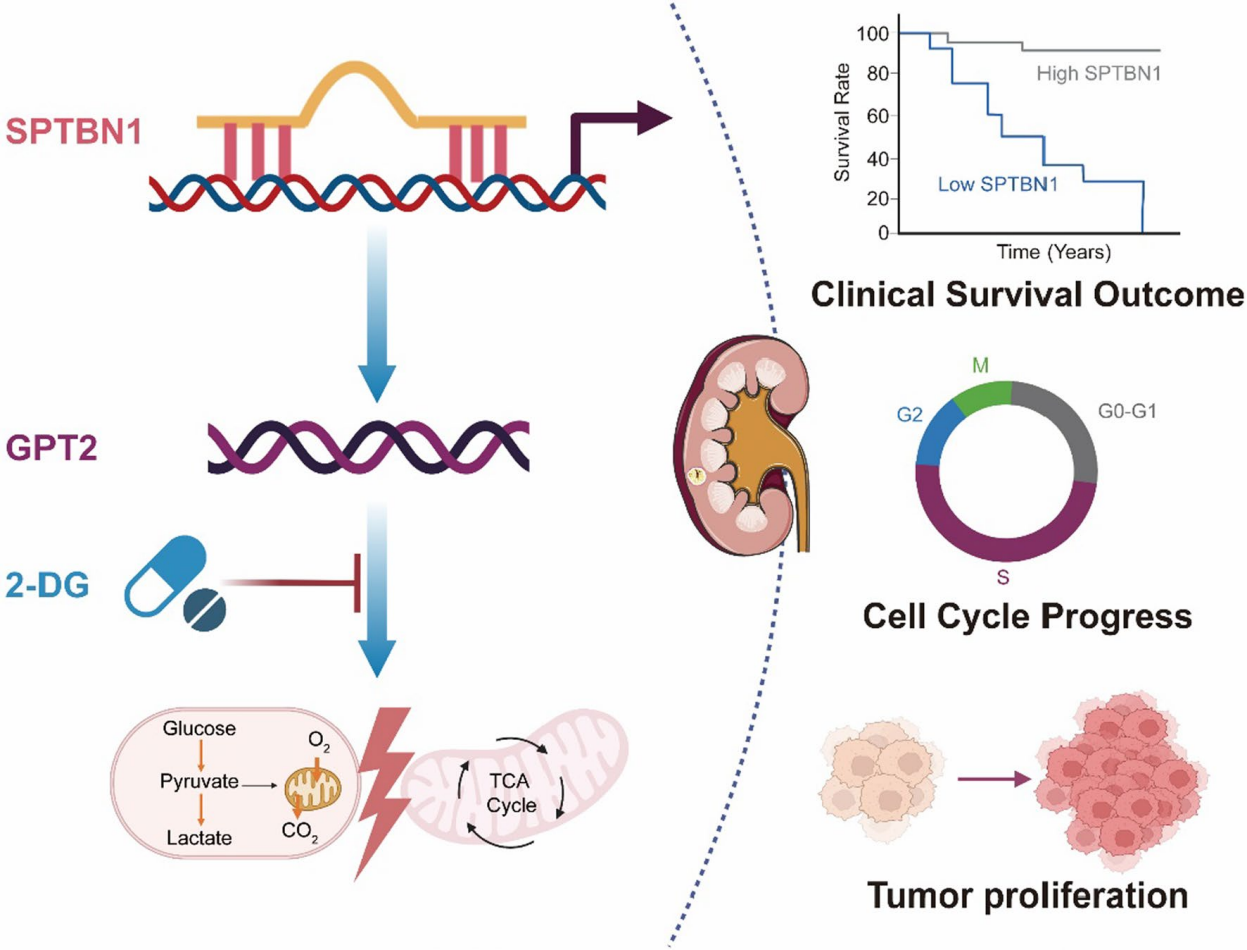
Schematic diagram of how SPTBN1 abrogates ccRCC progression through reprogramming glycolysis in a GPT2-dependent manner. Our research highlighted the mechanism of the SPTBN1-GPT2 axis in regulating glycolysis to abrogate ccRCC progression. These molecules and pathway may represent therapeutic targets to treat ccRCC

We investigated whether silencing SPTBN1 can reverse the malignant proliferation of ccRCC cells through directly modulating GPT2. CCK-8 assay revealed that GPT2 knockdown attenuated the oncogenic effect of SPTBN1 knockdown (Fig. 7D). Likewise, GPT2 knockdown abolished the ability of SPTBN1 in promoting colony formation (Fig. 7E). It was further validated that GPT2 knockdown could partly reduce ATP concentration, lactate production, and glucose uptake, implying that the glycolysis caused by SPTBN1 depletion in ccRCC cells (Fig. 7F, G). Collectively, SPTBN1 suppressed ccRCC development through degrading GPT2 to reprogram glyolysis (Fig. 8).

## Discussion

The prevalence of RCC has kept increasing worldwide in recent years [1, 3]. ccRCC is a typically heterogeneous disease with various molecular subtypes [66, 67]. scRNA-seq has proven as a potent tool for analyzing the heterogeneity of a tumor and the status of individual cells within tumor microenvironments [68–71]. In the present research, we for the first-time combined bulk RNA-seq and scRNA-seq to unveil the expression and distribution of Spectrin-family genes in ccRCC. We also teased out SPTBN1 as a novel ccRCC suppressor, as reflected by that its low expression could predict a poor survival and its overexpression could abrogate tumorigenesis.

Metabolic reprogramming is a hallmark as a tumor grows malignant [35]. Malignant cells can activate metabolic pathways, thus providing them nutrition enough to thrive and proliferate, despite that the patient it is taking dietary restrictions [72, 73]. ccRCC is characterized by the dysregulation of metabolic pathways, especially glycolytic pathway [31]. However, the molecular mechanism underlying metabolic reprogramming remains to be further explored. Glycolysis is closely involved in cancer proliferation, metabolism, migration and many another malignant processes [74–76]. Here, we found that SPTBN1 suppressed ccRCC progression through blocking glycolysis-related pathways involving PKM1/2, LDHA, HKII, and PFKP.

We further validated the mechanism of SPTBN1 in ccRCC glycolysis. Previous studies have demonstrated that SPTBN1 regulates SOCS3 at the transcriptional level through the TGF-β/Smads2/3 pathway [26]. Lin et al. have proven that SPTBN1 can induce the ubiquitination and degradation of p65 by means of SOCS1[22]. To investigate the mechanism by which SPTBN1 regulates GPT2, we used the online database of “RNAct” and confirmed SPTBN1 as an RNA-binding protein (RBP) [64, 65]. Then, we selected GPT2 as a direct downstream target of SPTBN1. Our research revealed that SPTBN1 could function as an RBP to regulate the mRNA stability of target genes. Based on our RNA-seq data, we found that SPTBN1 could bind to the 3’UTR in GPT2 mRNA to reduce its stability.

Glutamic pyruvate transaminase (GPT) participates in numerous cellular processes, primarily through transamination between alanine and α-ketoglutarate (α-KG) to generate pyruvate and glutamate [77, 78]. Several studies have discovered the implication of GPT2 in glucose metabolism and homeostasis [79]. For instance, GPT2 promotes tumorigenesis of breast cancer by activating sonic Hedgehog signaling [80], and abrogating GPT2 in triple-negative breast cancer can inhibit tumor growth and promotes autophagy [81]. Our findings elucidated that SPTBN1 interacted with GPT2 in a negative manner in both ccRCC tissues and cell lines, and that GPT2 could promote ccRCC cells proliferation in vitro via boosting glycolysis. These suggest GPT2 as a direct downstream molecule of SPTBN1 in the glycolytic pathway. Silencing GPT2 could partly reverse the malignant proliferation and the activation of glycolysis pathway caused by SPTBN1 deletion, further supporting the role of SPTBN1-GPT2-mediated glycolysis in promoting ccRCC oncogenesis. However, our findings need more ccRCC patient cohorts to verify its feasibility and constancy.

## Conclusions

SPTBN1 is significantly down-regulated in ccRCC. SPTBN1 knockdown promotes ccRCC progression via activating GPT2-dependent glycolysis. SPTBN1 may serve as a therapeutic target for the treatment of ccRCC.

## Supplementary Information

**Additional file 1: Figure S1.** Single-cell RNA-seq revealed the distribution of Spectrin-family genes. (A). Quality control of single-cell RNA-seq samples (Nine ccRCC sample). The number of gene expressions in each cell, the sum of gene expressions, and the percentage of mitochondrial genes were illustrated. (B). The correlation of number of genes in the cells with the sum of gene expression and the percentage of mitochondrial genes. (C). 3000 hypervariable genes from all the genes shown in red and the top 10 hypervariable genes. (D). Distribution of different clusters in ccRCC tissues obtained by UMAP algorithm. (E). Heatmap showed the results of the cell cluster obtained by cell marker gene annotation were consistent with those obtained by “singleR” package annotation. **Figure S2.** Univariate and multivariate cox regression to assess the prognosis value of Spectrin-family genes. (A). Univariate and multivariate cox regression between Spectrin-family genes and overall survival (OS). (B). Univariate and multivariate cox regression between Spectrin-family genes and disease-specific survival (DSS). (C). Univariate and multivariate cox regression between Spectrin-family genes and progression survival interval (PFI). **Figure S3.** Correlation and function enrichment of Spectrin-family genes. (A). The expression correlation analysis of Spectrin-family genes. (B). Functional enrichment demonstrated that Spectrin-family genes were mainly involved in the interaction between L1 and Ankyrins. (*: P<0.05; **: P<0.01). **Figure S4.** Identification of SPTBN1 expression level in the TCGA and CCLE pan-cancer dataset. (A-B). Pan-cancer expression level of SPTBN1 form TCGA database (A) and CCLE database (B). (ns: no significant; *: P<0.05; **: P<0.01; ***:P<0.001). **Figure S5.** The expression level of SPTBN1 in GEO datasets and IHC staining. (A-F). The expression level of SPTBN1 in GSE40435 cohort (A), GSE53757 cohort (B), GSE6344 cohort (C), GSE46699 cohort (D), GSE105261 cohort (E) and GSE66270 cohort (F). (G). IHC staining revealed SPTBN1 was down-regulated in ccRCC tissue compared with adjacent normal renal tissue. **Figure S6.** The protein expression level of SPTBN1 among different clinical grades ccRCC patients. (A). The protein expression level of SPTBN1 among different grade ccRCC patients in CPTAC cohort. (B). The protein expression level of SPTBN1 among different grade ccRCC patients in FUSCC-ccRCC cohort among Chinese ccRCC patients. (**: P<0.05). **Figure S7.** The correlation between SPTBN1 and survival outcomes in different clinicopathological characteristics subgroups. (A). The correlation between SPTBN1 and survival outcomes among age<=60 and age>60 subgroups. (B). The correlation between SPTBN1 and survival outcomes among female and male subgroups. (C). The correlation between SPTBN1 and survival outcomes among stage I-II and stage III-IV subgroups. (D). The correlation between SPTBN1 and survival outcomes among T1-T2 and T3-T4 subgroups. **Figure S8.** Validation of SPTBN1 expression level after SPTBN1-knockdown and SPTBN1-overexpressing by qRT-PCR and WB. (A). qRT-PCR and WB assays validated the expression of SPTBN1 after knockdown SPTBN1. (B). qRT-PCR and WB assays validated the expression of SPTBN1 after overexpression SPTBN1. (C). qRT-PCR and WB assays validated the expression of GPT2 after knockdown GPT2. (**: P<0.05). **Figure S9.** Transwell cell migration and invasion assays after SPTBN1-knockdown and SPTBN1-overexpressing of RCC cells. (**: P<0.05). **Figure S10.** IHC staining revealed GPT2 was up-regulated in ccRCC tissue compared with adjacent normal renal tissue. **Table S1.** Oligonucleotide sequences used in this research

## Data Availability

All data generated or analyzed during this study were included either in this article methods section or in the Supplemental Information. Other data that support the findings of this study are available from the corresponding author upon reasonable request.
