# Supplementary material for: SPTBN1 abrogates renal clear cell carcinoma progression via glycolysis reprogramming in a GPT2-dependent manner
Source: J Transl Med. 2022 Dec 16;20:603. doi: 10.1186/s12967-022-03805-w (PMC9756479; doi:10.1186/s12967-022-03805-w)
Supplement: Supplementary file 1 — Additional file 1: Figure S1. Single-cell RNA-seq revealed the distribution of Spectrin-family genes. (A). Quality control of single-cell RNA-seq samples (Nine ccRCC sample). The number of gene expressions in each cell, the sum of gene expressions, and the percentage of mitochondrial genes were illustrated. (B). The correlation of number of genes in the cells with the sum of gene expression and the percentage of mitochondrial genes. (C). 3000 hypervariable genes from all the genes shown in red and the top 10 hypervariable genes. (D). Distribution of different clusters in ccRCC tissues obtained by UMAP algorithm. (E). Heatmap showed the results of the cell cluster obtained by cell marker gene annotation were consistent with those obtained by “singleR” package annotation. Figure S2. Univariate and multivariate cox regression to assess the prognosis value of Spectrin-family genes. (A). Univariate and multivariate cox regression between Spectrin-family genes and overall survival (OS). (B). Univariate and multivariate cox regression between Spectrin-family genes and disease-specific survival (DSS). (C). Univariate and multivariate cox regression between Spectrin-family genes and progression survival interval (PFI). Figure S3. Correlation and function enrichment of Spectrin-family genes. (A). The expression correlation analysis of Spectrin-family genes. (B). Functional enrichment demonstrated that Spectrin-family genes were mainly involved in the interaction between L1 and Ankyrins. (*: P<0.05; **: P<0.01). Figure S4. Identification of SPTBN1 expression level in the TCGA and CCLE pan-cancer dataset. (A-B). Pan-cancer expression level of SPTBN1 form TCGA database (A) and CCLE database (B). (ns: no significant; *: P<0.05; **: P<0.01; ***:P<0.001). Figure S5. The expression level of SPTBN1 in GEO datasets and IHC staining. (A-F). The expression level of SPTBN1 in GSE40435 cohort (A), GSE53757 cohort (B), GSE6344 cohort (C), GSE46699 cohort (D), GSE105261 cohort (E) and GSE66 [file 12967_2022_3805_MOESM1_ESM.docx]

**Supplementary materials:**

**Figure S1: Single-cell RNA-seq revealed the distribution of Spectrin-family genes.**

(A). Quality control of single-cell RNA-seq samples (Nine ccRCC sample). The number of gene expressions in each cell, the sum of gene expressions, and the percentage of mitochondrial genes were illustrated. (B). The correlation of number of genes in the cells with the sum of gene expression and the percentage of mitochondrial genes. (C). 3000 hypervariable genes from all the genes shown in red and the top 10 hypervariable genes. (D). Distribution of different clusters in ccRCC tissues obtained by UMAP algorithm. (E). Heatmap showed the results of the cell cluster obtained by cell marker gene annotation were consistent with those obtained by “singleR” package annotation.


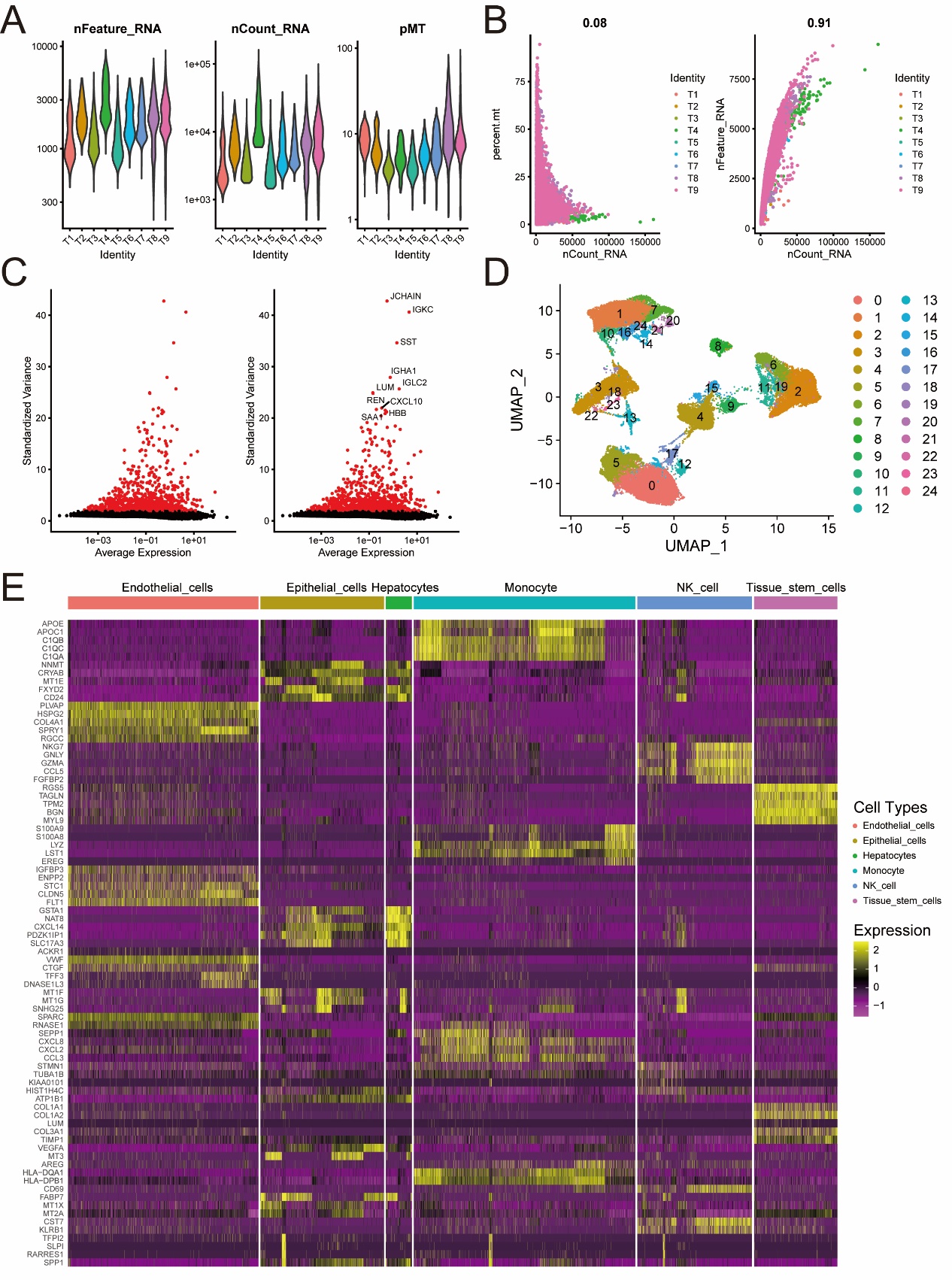


**Figure S2: Univariate and multivariate cox regression to assess the prognosis value of Spectrin-family genes.**

(A). Univariate and multivariate cox regression between Spectrin-family genes and overall survival (OS). (B). Univariate and multivariate cox regression between Spectrin-family genes and disease-specific survival (DSS). (C). Univariate and multivariate cox regression between Spectrin-family genes and progression survival interval (PFI).


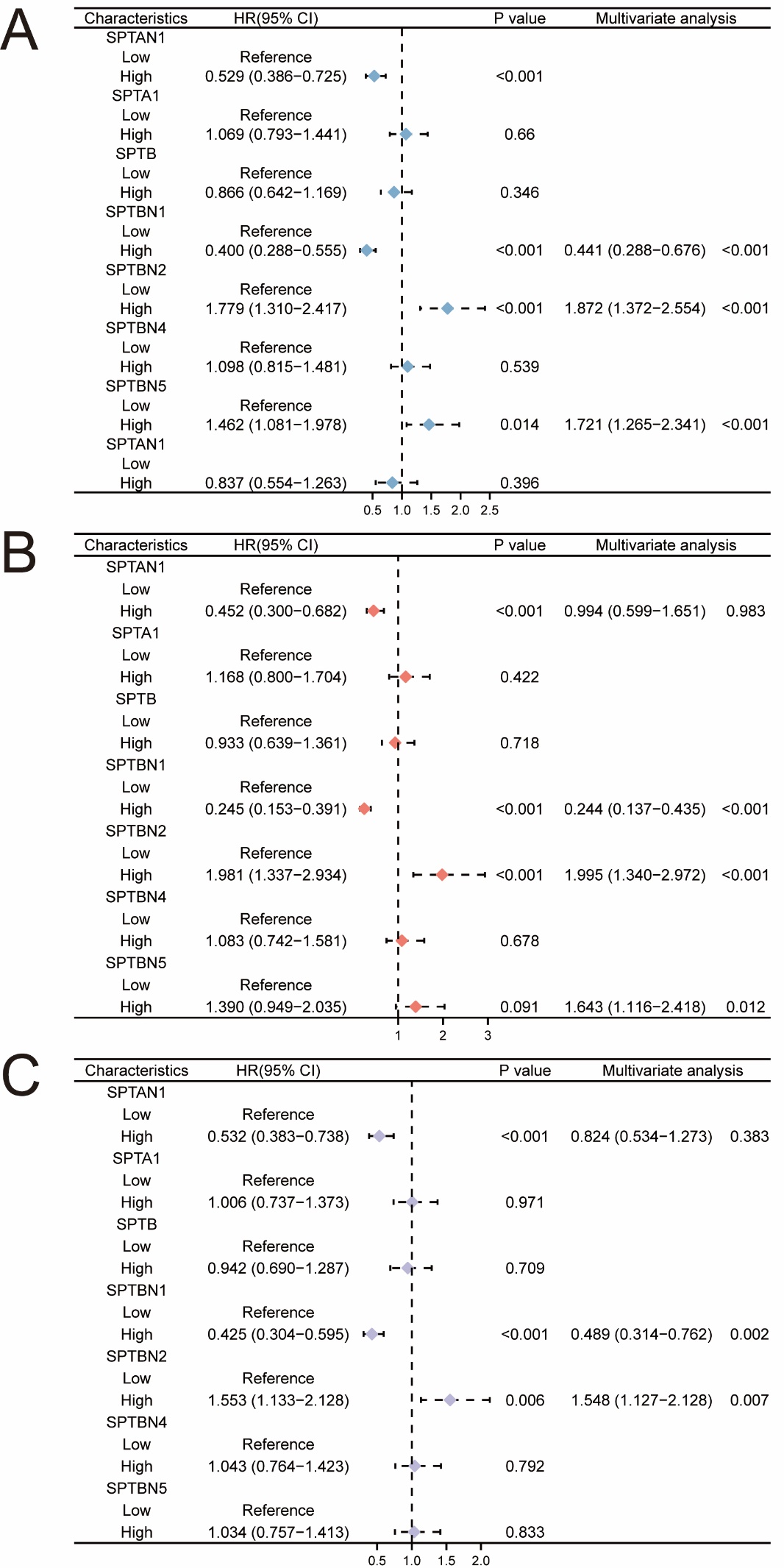


**Figure S3: Correlation and function enrichment of Spectrin-family genes.**

(A). The expression correlation analysis of Spectrin-family genes. (B). Functional enrichment demonstrated that Spectrin-family genes were mainly involved in the interaction between L1 and Ankyrins. (*: *P*<0.05; **: *P*<0.01)


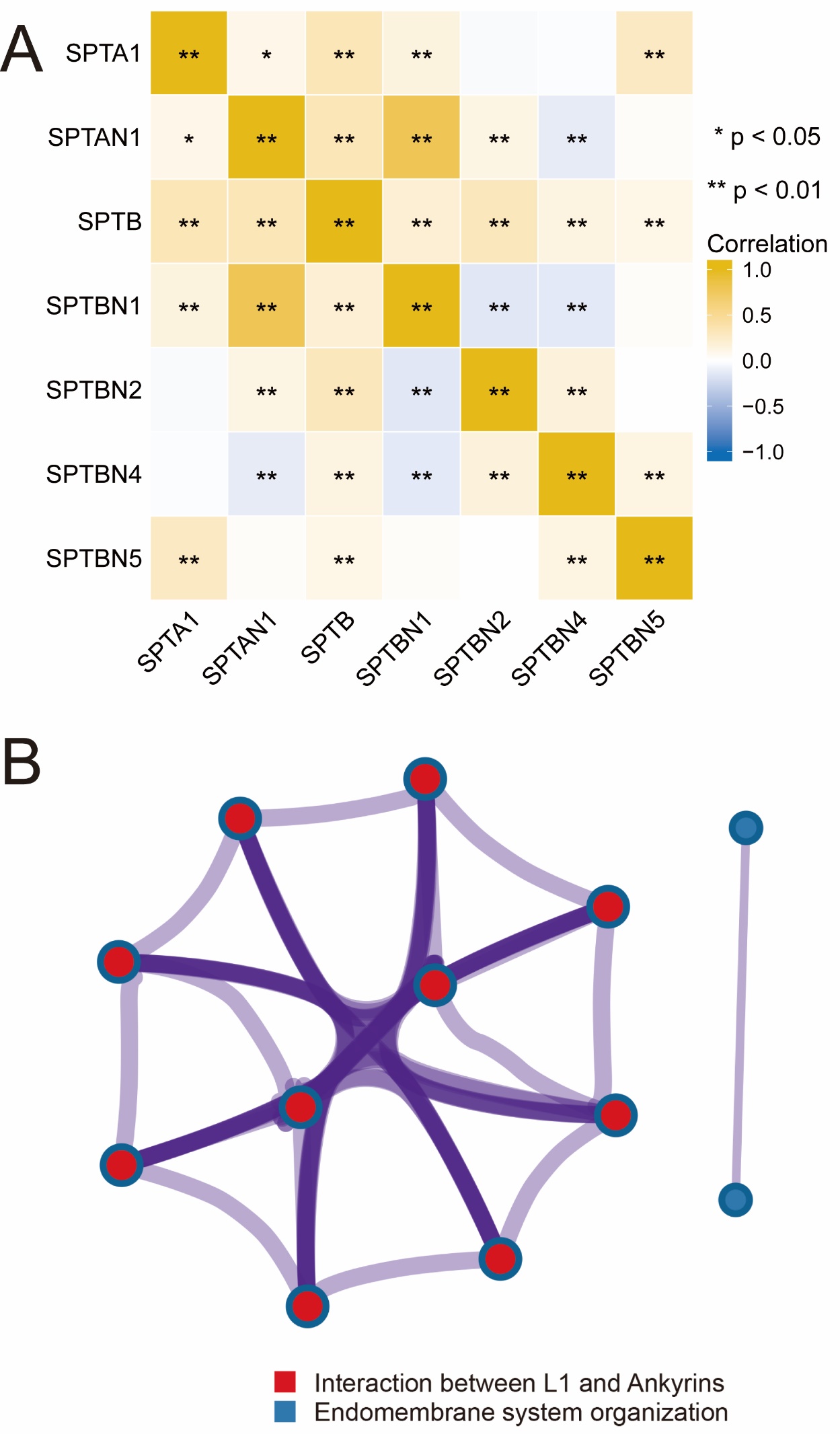


**Figure S4: Identification of SPTBN1 expression level in the TCGA and CCLE pan-cancer dataset.**

(A-B). Pan-cancer expression level of SPTBN1 form TCGA database (A) and CCLE database (B). (ns: no significant; *: *P*<0.05; **: *P*<0.01; ***:*P*<0.001)


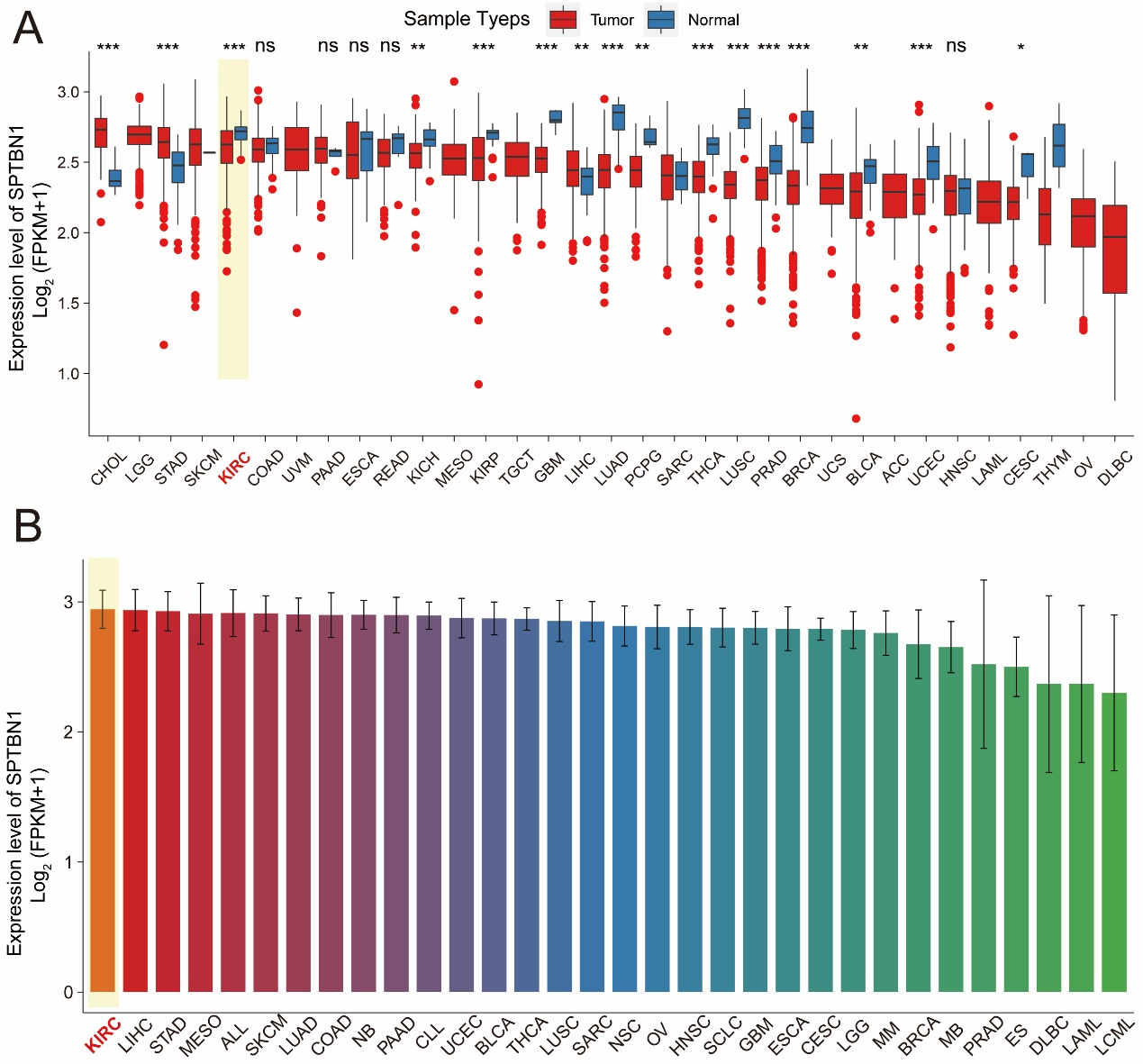


**Figure S5: The expression level of SPTBN1 in GEO datasets and IHC staining.**

(A-F). The expression level of SPTBN1 in GSE40435 cohort (A), GSE53757 cohort (B), GSE6344 cohort (C), GSE46699 cohort (D), GSE105261 cohort (E) and GSE66270 cohort (F). (G). IHC staining revealed SPTBN1 was down-regulated in ccRCC tissue compared with adjacent normal renal tissue.


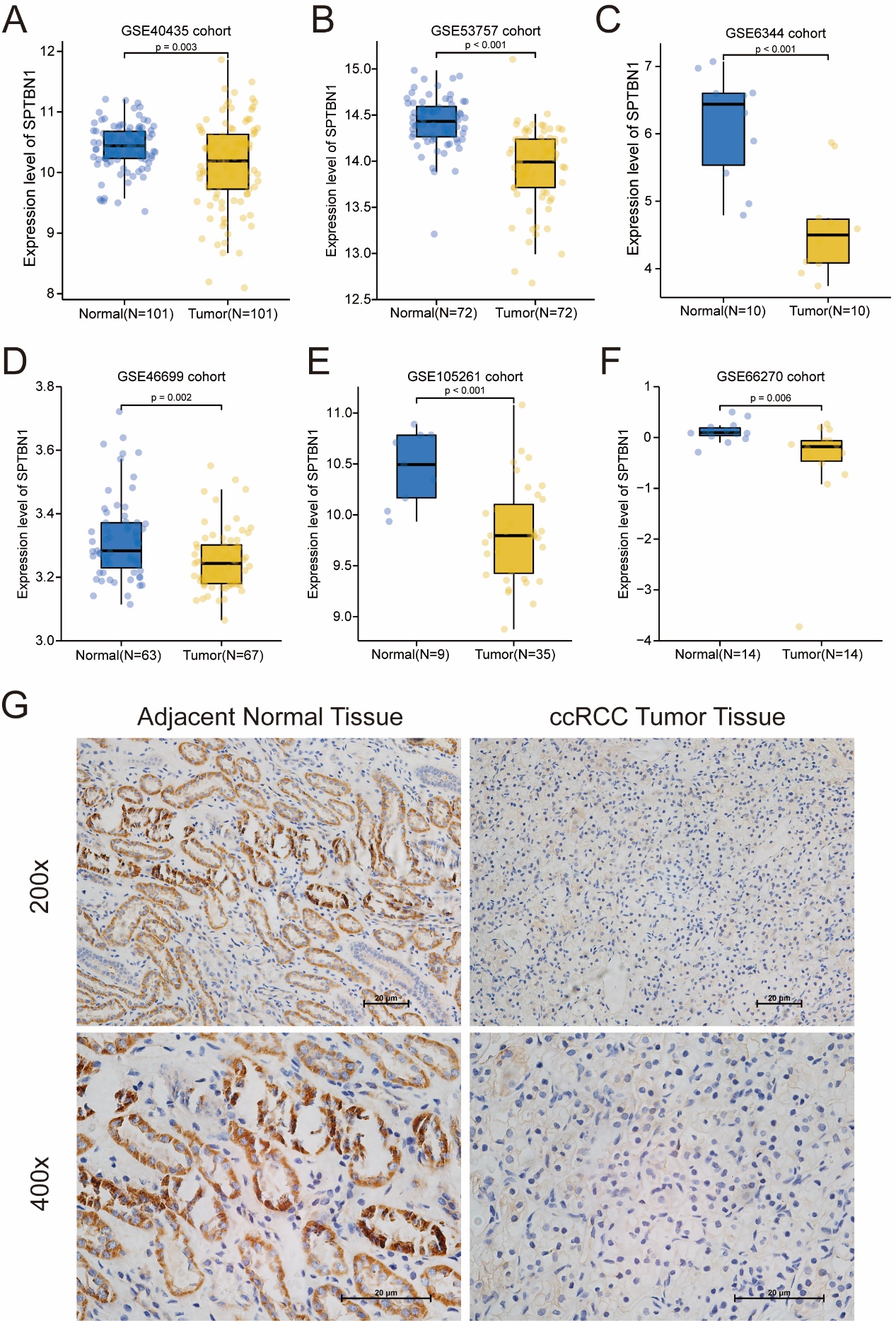


**Figure S6: The protein expression level of SPTBN1 among different clinical grades ccRCC patients.**

(A). The protein expression level of SPTBN1 among different grade ccRCC patients in CPTAC cohort. (B). The protein expression level of SPTBN1 among different grade ccRCC patients in FUSCC-ccRCC cohort among Chinese ccRCC patients. (**: *P*<0.05)


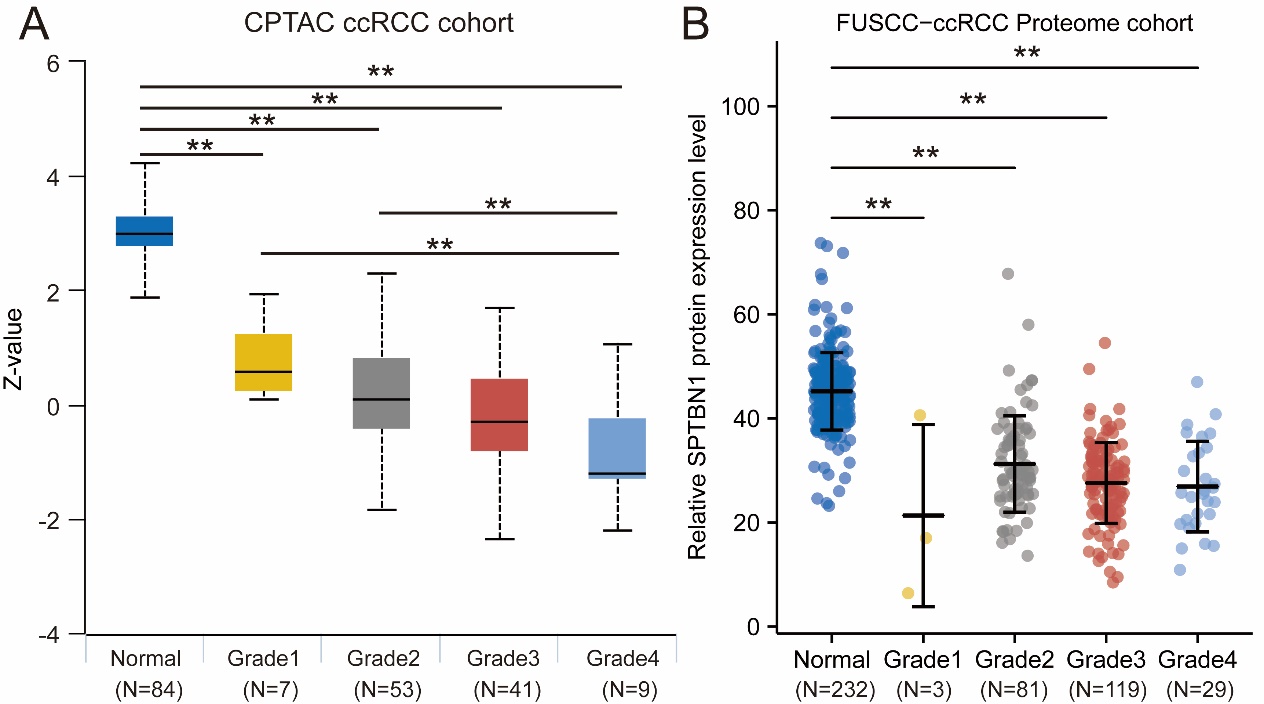


**Figure S7: The correlation between SPTBN1 and survival outcomes in different clinicopathological characteristics subgroups.**

(A). The correlation between SPTBN1 and survival outcomes among age<=60 and age>60 subgroups. (B). The correlation between SPTBN1 and survival outcomes among female and male subgroups. (C). The correlation between SPTBN1 and survival outcomes among stage I-II and stage III-IV subgroups. (D). The correlation between SPTBN1 and survival outcomes among T1-T2 and T3-T4 subgroups.


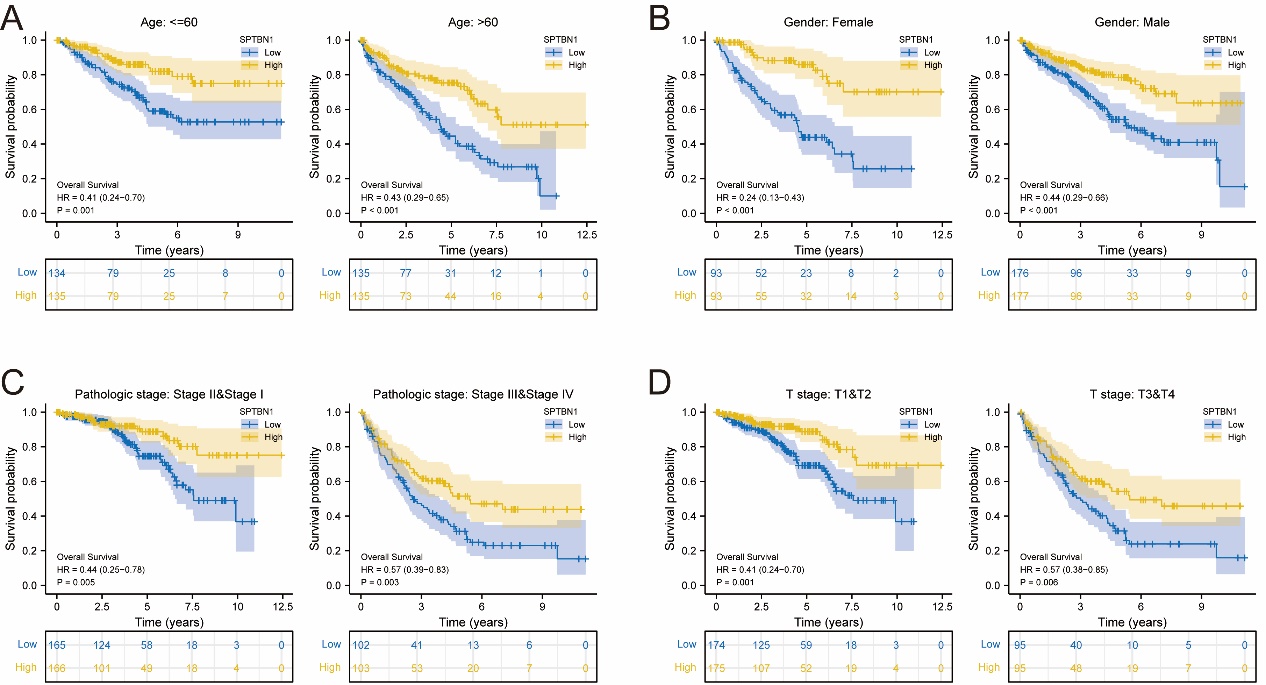


**Figure S8: Validation of SPTBN1 expression level after SPTBN1-knockdown and SPTBN1-overexpressing by qRT-PCR and WB.**

(A). qRT-PCR and WB assays validated the expression of SPTBN1 after knockdown SPTBN1. (B). qRT-PCR and WB assays validated the expression of SPTBN1 after overexpression SPTBN1. (C). qRT-PCR and WB assays validated the expression of GPT2 after knockdown GPT2. (**: *P*<0.05)


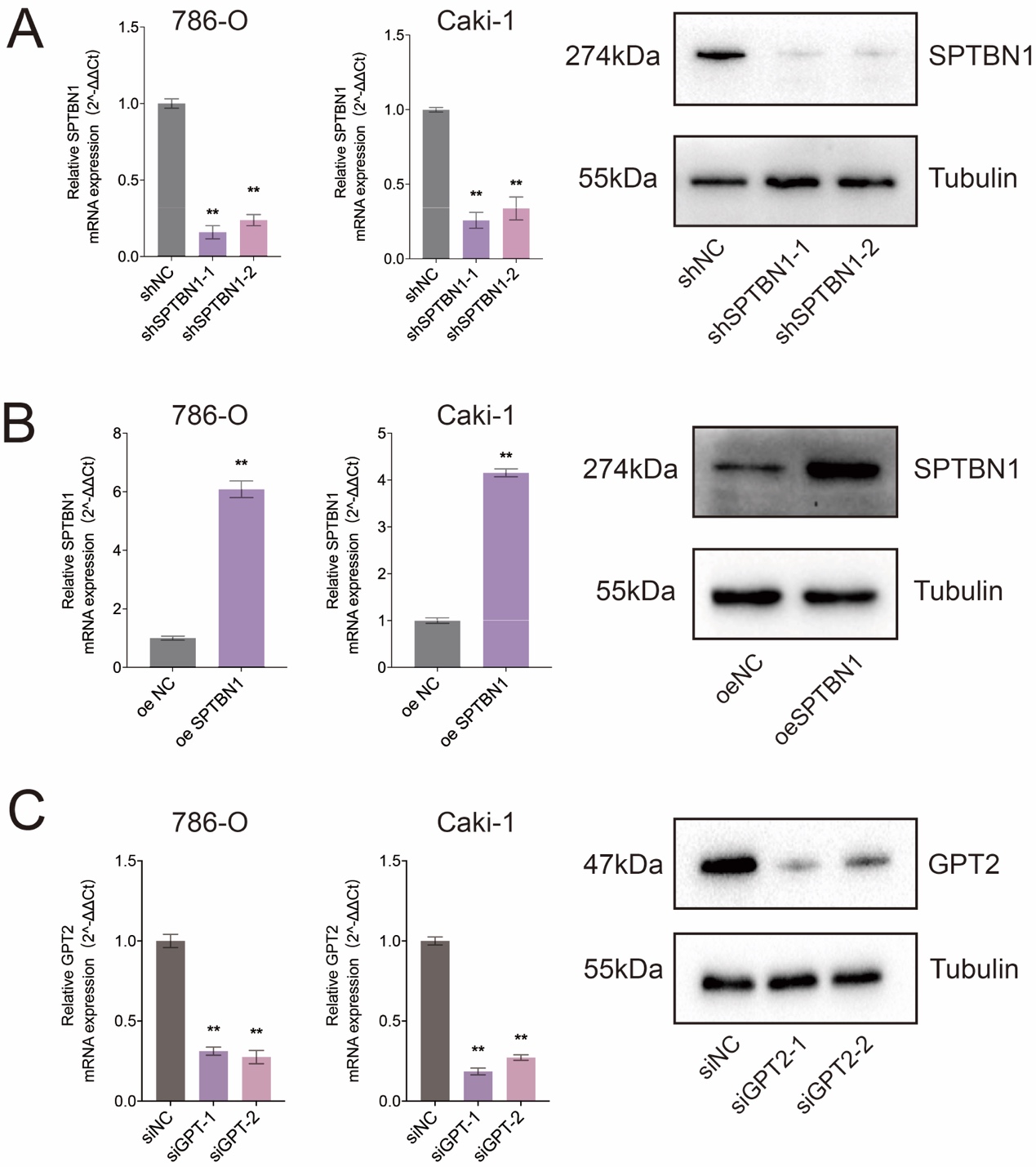


**Figure S9: Transwell cell migration and invasion assays after SPTBN1-knockdown and SPTBN1-overexpressing of RCC cells. (**: *P*<0.05)**


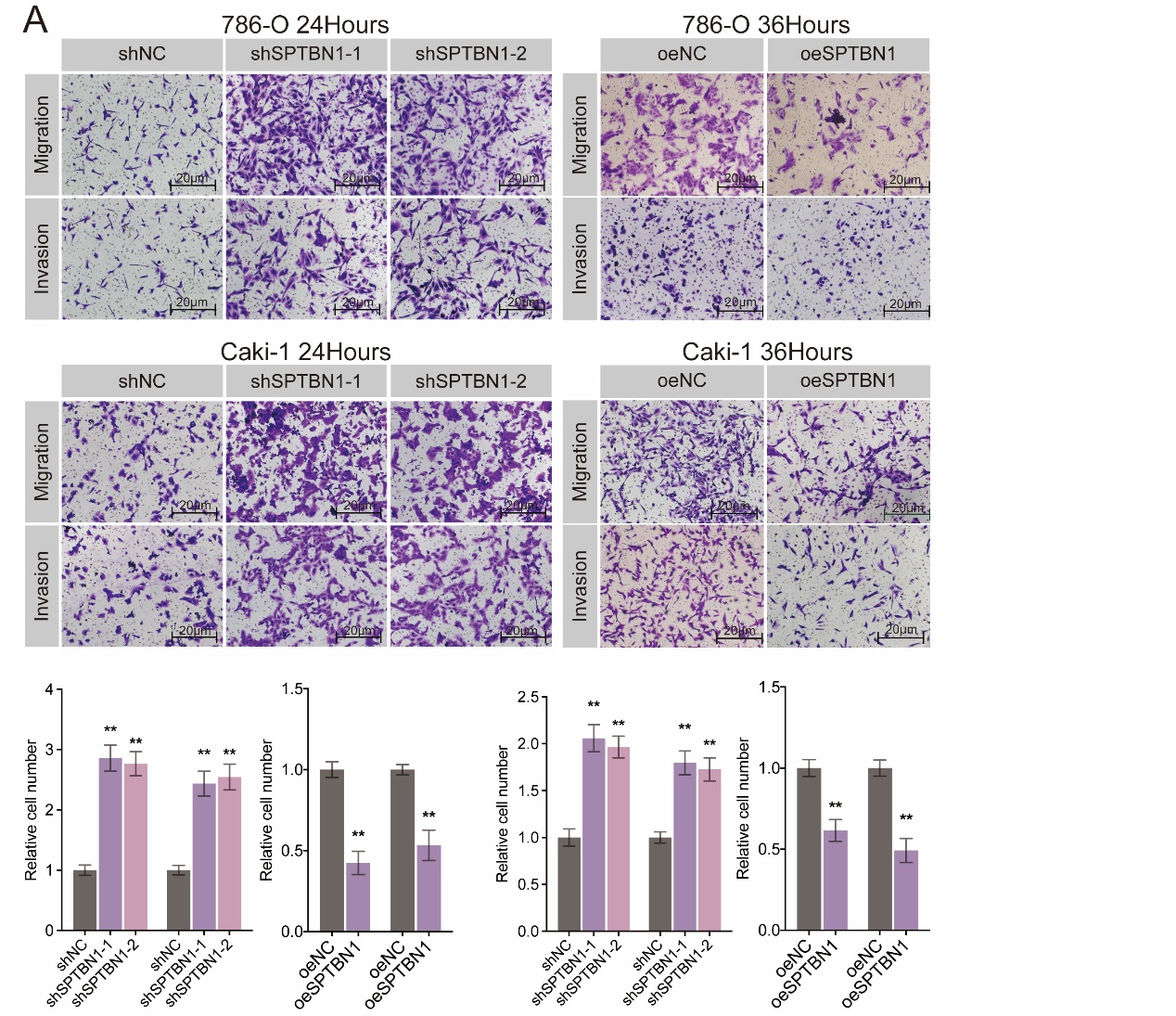


**Figure S10: IHC staining revealed GPT2 was up-regulated in ccRCC tissue compared with adjacent normal renal tissue.**


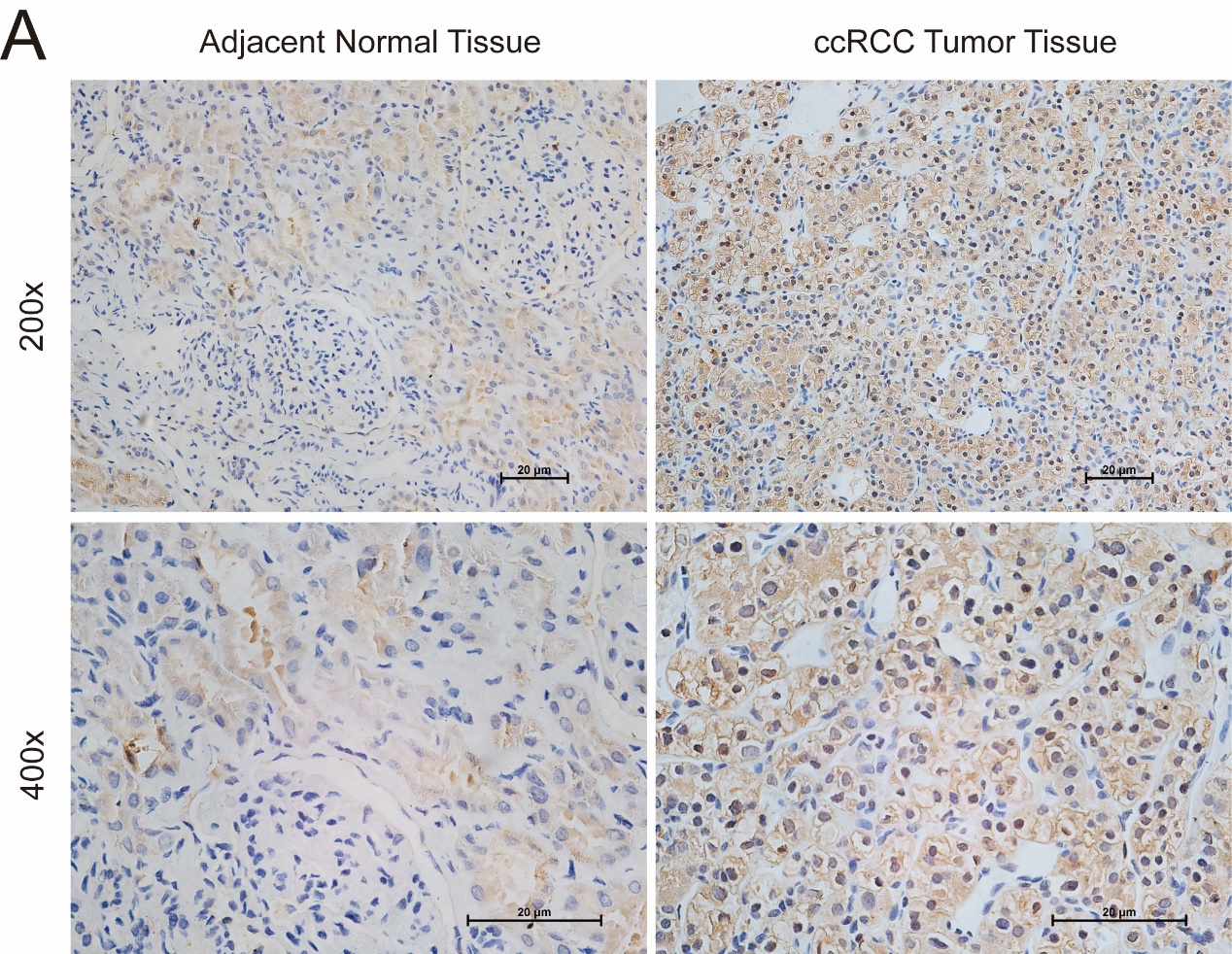


**Table S1 Oligonucleotide sequences used in this research**

| Primers | Sequences | |
| --- | --- | --- |
| siNC | Forward | UUCUCCGAACGUGUCACGUTT |
|  | Reverse | ACGUGACAGGUUCGGAGAATT |
| siGPT2-1 | Forward | GCUUCCCAGAAGAUGCUAATT |
|  | Reverse | UUAGCAUCUUCUGGGAAGCTT |
| siGPT2-2 | Forward | CCUAAGGUGCUCUGCAUAATT |
|  | Reverse | UUAUGCAGAGCACCUUAGGTT |
| siGPT2-3 | Forward | GCUGACGGAAGACCUGUUUTT |
|  | Reverse | AAACAGGUCUUCCGUCAGETT |
| siNC | Forward | GGAACGUGGAGUGCAGAUCUUAAUC |
|  | Reverse | GAUUAAGAUCUGCACUCCACGUUCC |
| siSPTBN1-1 | Forward | GGAAUUGCAGAGGACGUCUAGUAUC |
|  | Reverse | GAUACUAGACGUCCUCUGCAAUUCC |
| siSPTBN1-2 | Forward | ACCUUCGAGAUGGACGGAUGCUCAU |
|  | Reverse | AUGAGCAUCCGUCCAUCUCGAAGGU |
| SPTBN1 | Forward | GGGGTCCCATGACATCGTG |
|  | Reverse | CCGGTGTTTGTGTATCAGTGC |
| GPT2 | Forward | GTGATGGCACTATGCACCTAC |
|  | Reverse | TTCACGGATGCAGTTGACACC |
| PKM | Forward | ATGTCGAAGCCCCATAGTGAA |
|  | Reverse | TGGGTGGTGAATCAATGTCCA |
| HKII | Forward | GAGCCACCACTCACCCTACT |
|  | Reverse | CCAGGCATTCGGCAATGTG |
| PFKFB3 | Forward | TTGGCGTCCCCACAAAAGT |
|  | Reverse | AGTTGTAGGAGCTGTACTGCTT |
| ENO2 | Forward | AGCCTCTACGGGCATCTATGA |
|  | Reverse | TTCTCAGTCCCATCCAACTCC |
| SLC2A1 | Forward | GGCCAAGAGTGTGCTAAAGAA |
|  | Reverse | ACAGCGTTGATGCCAGACAG |
| PFKP | Forward | GCATGGGTATCTACGTGGGG |
|  | Reverse | CTCTGCGATGTTTGAGCCTC |
| LDHA | Forward | ATGGCAACTCTAAAGGATCAGC |
|  | Reverse | CCAACCCCAACAACTGTAATCT |
| β-actin | Forward | ATGACTTAGTTGCGTTACACC |
|  | Reverse | GACTTCCTGTAACAACGCATC |
